# Comparative analysis of daily global solar radiation prediction using deep learning models inputted with stochastic variables

**DOI:** 10.1038/s41598-025-95281-7

**Published:** 2025-03-28

**Authors:** Amit Kumar Yadav, Raj Kumar, Meizi Wang, Gusztáv Fekete, Tej Singh

**Affiliations:** 1 School of Computer Science and Artificial Intelligence, SR University, Warangal, Telangana 506371 India; 2 Department of Mechanical Engineering, Gachon University, 1342 Seongnamdaero, Sujeong-gu, Seongnam, 13120 South Korea; 3 Department of Biomedical Engineering, Faculty of Engineering, The Hong Kong Polytechnic University, Hong Kong, China; 4 Department of Materials Science and Technology, Széchenyi István University, Győr, 9026 Hungary; 5 Faculty of Informatics, Eötvös Loránd University, Budapest, 1117 Hungary

**Keywords:** Solar radiation, Daily prediction, Modular neural network, Long short-term memory, Transformer model, Artificial neural network, Engineering, Mathematics and computing

## Abstract

Photovoltaic power plant outputs depend on the daily global solar radiation (DGSR). The main issue with DGSR data is its lack of precision. The potential unavailability of DGSR data for several sites can be attributed to the high cost of measuring instruments and the intermittent nature of time series data due to equipment malfunctions. Therefore, DGSR prediction research is crucial nowadays to produce photovoltaic power. Different artificial neural network (ANN) models will give different DGSR predictions with varying levels of accuracy, so it is essential to compare the different ANN model inputs with various sets of meteorological stochastic variables. In this study, radial basis function neural network (RBFNN), long short-term memory neural network (LSTMNN), modular neural network (MNN), and transformer model (TM) are developed to investigate the performances of these algorithms for the DGSR prediction using different combinations of meteorological stochastic variables. These models employ five stochastic variables: wind speed, relative humidity, minimum, maximum, and average temperatures. The mean absolute relative error for the transformer model with input variables as average, maximum, and minimum temperatures is 1.98. ANN models outperform traditional models in predictive accuracy.

**Supplementary Information:**

The online version contains supplementary material available at 10.1038/s41598-025-95281-7.

## Introduction

Solar radiation is now used more frequently and is crucial for photovoltaic systems and solar thermal applications due to rising conventional fuel costs, energy needs, and environmental consciousness^1,2^. For many sites, the solar radiation data needs to be dependable and simple to get. Unfortunately, many developing nations like India cannot access this data at different latitudes due to a lack of measurement equipment. In solar energy research, finding relationships between global solar radiation and climatic factors has been complex. Although several conventional regression models have been created for solar radiation estimation, the demand for convenient and accurate estimate methods remains strong^3–5^. Various artificial neural network algorithms have been employed by multiple researchers for solar radiation prediction across the world^6,7^. The daily global solar radiation (DGSR) for the Iranian city of Dezful was predicted by Behrang et al.^8^ using “multi-layer perceptron” and “radial basis function neural networks” (RBFNN) as inputs. The variables utilized in this prediction were the day of the year (D), evaporation, average temperature (T), relative humidity (RH), wind speed (WS) and sunshine hours (SH). The correlation value for the multi-layer perceptron model using RH, T, WS, D, and SH as input variables is 99.57%. In comparison, 99.45% of correlation was recorded for the RBFNN model using SH, D, and T as input variables. Benghanem and Mellit^9^ presented D, RH, SH, and T as inputs to RBFNN to predict DGSR. RBFNN prediction over conventional regression models is recommended since the correlation is 98.80%. The RBFNN model with SH and T was discovered to have a superior correlation value (98.80%) and produce better outcomes than multi-layer perceptron and traditional regression models. Benghanem et al.^10^ created six artificial neural network (ANN) models to estimate global solar radiation in Al-Madinah (Saudi Arabia) using different SH, T, D, and RH permutations as inputs. T and D may be used for prediction when SH is unavailable, and the R-value of an ANN model built with SH and T as input rises to 97.65%. Yacef et al.^11^ used the automated relevance determination approach to evaluate different input variables to the ANN model and declared that SH and T are the most pertinent factors for DGSR prediction. The Bayesian Neural Network produces more thorough results than empirical models and neural networks. Yadav et al.^12,13^ chose pertinent input factors for solar radiation prediction using Waikato Environment for Knowledge Analysis (WEKA) and Rapid Miner. Meenal and Selvakumar^14^ used WEKA for relevant input selection and discovered that in comparison to RH, SH, latitude, month, and maximum temperature (T_max_) are the most influential variables. Liu et al.^15^ describe ensemble learning methods incorporating data from many sources to provide reliable and consistent predictions.

Tree-based machine learning models and support vector machine (SVM) were implemented to daily horizontal solar radiation prediction by Fan et al.^16^, and it was discovered that SVM offered greater accuracy and generalization potential. Six input combinations of DGSR, SH, T_max_, minimum temperature (T_min_) and RH were used in the analysis. Wu et al.^17^ introduced Bayesian additive regression trees and claimed that the prediction of diffuse solar radiation has the lowest error possible. Gao et al.^18^ employed a deep generative model based on long short-term memory neural network (LSTMNN) and found that it can boost accuracy by 7.7% compared to a traditional regression LSTMNN model. Zhao et al.^19^ provided different machine-learning algorithms and discovered that using data from satellites and ground meteorological stations as input led to a model with greater simulation accuracy. The study conducted by Goliatt and Yaseen^20^ investigates the application of computational intelligence models that integrate multi-adaptive regression splines and extreme gradient boosting (XGBoost) with covariance matrix adaptive evolution strategies to develop robust predictive models for solar radiation at Gaoua, Dori, Po, and Boromo meteorological stations in Burkina Faso. The findings indicated that the suggested framework consistently improved prediction accuracy by 39.8%, 4.9%, 44.5%, and 17.2% relative to current methodologies. The study by Abdallah et al.^21^ combines a “multi-functional recurrent fuzzy neural network” (MFRFNN) and variational mode decomposition (VMD) to predict DGSR across two meteorological stations (Växjö and Lund) in Sweden. The hybrid VMD-MFRFNN model was evaluated against MFRFNN, QRF (quantile regression forests), RF (random forest), M5 Tree (M5T), and XGBoost. The proposed VMD-MFRFNN approach exhibited optimal performance with a reduced number of input variables, specifically, T, sunlight duration, T_max_, T_min_, RH, and WS at Växjö meteorological station, as well as T, T_min_, WS, and T_max_ at Lund meteorological station for DGSR prediction. Overall, the study found that when all meteorological variables (T, T_min_, WS, T_max_ sunlight duration, RH, maximum potential sunshine duration) are combined, the MFRFNN model provides the best average DGSR predictions. Furthermore the studies reported by different authors with ANN models and prediction accuracy are shown in Table 1.

**Table 1:** Comparative analysis of present work error with published work.

Studies	Model	Highlights
Present	RBFNN, LSTMNN, MNN and TM	MARE of TM is 1.98
Azizi et al.^22^	MLP, LSTM, GRU, CNN, and CNN-LSTM	RMSE in solar radiation prediction is 12.68 W/m^2^
Gbémou et al.^23^	Gaussian process regression, support vector regression, and artificial neural networks	LSTM, Gaussian process regression are considered as best model
Zhu et al.^24^	Decomposition technique, clustering technique, optimizer and CatBoost algorithm	*R* = 0.99, RMSE = 9.68, and MAE = 3.85.
Gallo et al.^25^	ConvLSTM	Normalized RMSE of 0.57 for 6-h ahead predictions
Sansine et al.^26^	PSO-XGBoost, PSO-LSTM, PSO-GBRT	PSO-LSTM predict best with MAE = 99.37 W/m^2^, RMSE = 154.84 W/m^2^, and R^2^ = 0.82
Hanif and Mi^27^	TM	TM enhance solar radiation forecasting accuracy

The most valuable inputs for prediction are T_max_, T_min_, and SH. T_max_, and T_min_ may also be used to predict locations where SH data are not obtained. A notable research need identified in the study by Yadav and Chandel^28^, Singla et al.^29^, and Alcántara et al.^30^ is the influence of diverse geographical and meteorological parameters on the forecast accuracy of ANN-based solar radiation models. In light of this research gap, sixteen models (RBFNN-1, RBFNN-2, RBFNN-3, RBFNN-4, LSTMNN-1, LSTMNN-2, LSTMNN-3, LSTMNN-4, MNN-1, MNN-2, MNN-3, MNN-4, TM-1, TM-2, TN-3, and TM-4) employing various input combinations were developed to predict DGSR on a horizontal surface in Hamirpur, a city situated in the Indian state of Himachal Pradesh within the western Himalayas. Input variables for RBFNN-1, LSTMNN-1, TM-1 and MNN-1 are RH, WS, T, T_max_, and T_min_. For RBFNN-2, LSTMNN-2, TM-2 and MNN-2, the inputs RH, T, T_max_, and T_min_ are utilized. For RBFNN-3, LSTMNN-3, TM-3 and MNN-3, the inputs WS, T, T_max_, and T_min_ are used. RBFNN-4, LSTMNN-4, TM-4 and MNN-4 all need the inputs T, T_max_, and T_min_. These models are evaluated in comparison to many traditional models.

This paper is organized as follows: “Related work” section provides related work in context with solar radiation prediction. Section 3 presents problem of solar radiation data and prediction. Section 4 provides methodology. Section 5 provides data collection. Section 6 provides details of results and discussions. Section 7 highlights conclusions.

## Related work

SR is important for PV system, climate modeling and solar energy planning. Different methods are developed for prediction of solar radiation varies from conventional to machine learning models. The literature survey of different methods comparative analysis is shown in Table 2.

**Table 2:** Literature survey of different models.

Study	Research objective	Models used	Highlights
Bamisile et al.^31^	Comparative analysis of eight different artificial intelligence models	Convolution neural network, ANN, LSTM, XGBoost, multiple linear regression, polynomial regression, decision tree regression, and RF regression.	Prediction of hourly solar radiation is more accurate in comparison to daily average and minute’s time step.
Escalona-Llaguno et al.^32^	Prediction of DGSR in Zacatecas, Mexico, using a diverse range of predictive models with different models	Evolutionary neural architecture search, convolution neural networks, recurrent neural networks and meta’s prophet.	Correlation coefficient ranged between 0.55 and 0.72, with RMSE values spanning from 20.05–20.54%, indicating moderate to good predictive accuracy.
Huang et al.^33^	Comparative analysis of 12 machine learning models for daily and monthly prediction of solar radiation.	Multiple linear regression, RF, RBFNN, XGBoost, k-nearest neighbor, decision tree, back-propagation neural network, extreme learning machine, SVM regression, Gaussian process regression, AdaBoost, Gradient boosting regression tree.	XGBoost and stacking model are the best models to predict solar radiation.
Nadeem et al.^34^	To predict DGSR for six different cities of Pakistan	RF regression, k-nearest neighbors, Gradient boosting regression, and SVM regression.	Data is stored using pyranometer sensor and microcontroller proving cost effective.
Yu et al.^35^	Performance computation of four machine learning models	Extreme learning machine, hybrid artificial neural networks with genetic algorithm models (GANN), generalized regression neural networks and RF.	Extreme learning machine and GANN give better result in comparison to other consider model.
Bamisile et al.^36^	Predictive performance of machine learning models is compared.	Deep learning models.	Regression value of 95.46% is obtained.
Paletta et al.^37^	Comparison of four commonly used deep learning architectures	Deep learning models.	20.4% is achieved on the test year.
Jiang and Zhu^38^	Feature selection technique	Pearson correlation between DGSR and each input parameter.	Informer’s predictive performance is excellent.

## Problem of solar radiation data and prediction

Pyranometers, solarimeters, pyro heliometers, etc., are used with a data gathering system to measure solar radiation. Due to their high cost, solar radiation measuring equipment cannot be installed at every site, which prevents the availability of measured solar radiation data for many sites globally. Additionally, due to the measurement equipment’s lack of precision, missing records in the data set have been detected. Though “National Aeronautics and Space Administration” (NASA) database values for solar radiation are easily accessible for different locations, they lack accuracy, showing higher values than ground-measured solar radiation^39^. The maps developed by the “National Renewable Energy Laboratory” (NREL), U.S.A., give different ranges of solar radiation, which is higher due to the utilization of the “State University of New York” (SUNY) model. The estimated values by the SUNY model are higher in comparison to NASA and ground-measured values. In addition, the mapping values do not give distinct values of solar radiation, which is essential for optimum sizing and tilt angle calculations. Therefore, conventional and ANN methods are mainly used for prediction at different locations where measuring instruments are not available to solve the problem of solar radiation data’s non-availability.

## Methodology

### Radial basis function neural network (RBFNN)

**Fig. 1:**
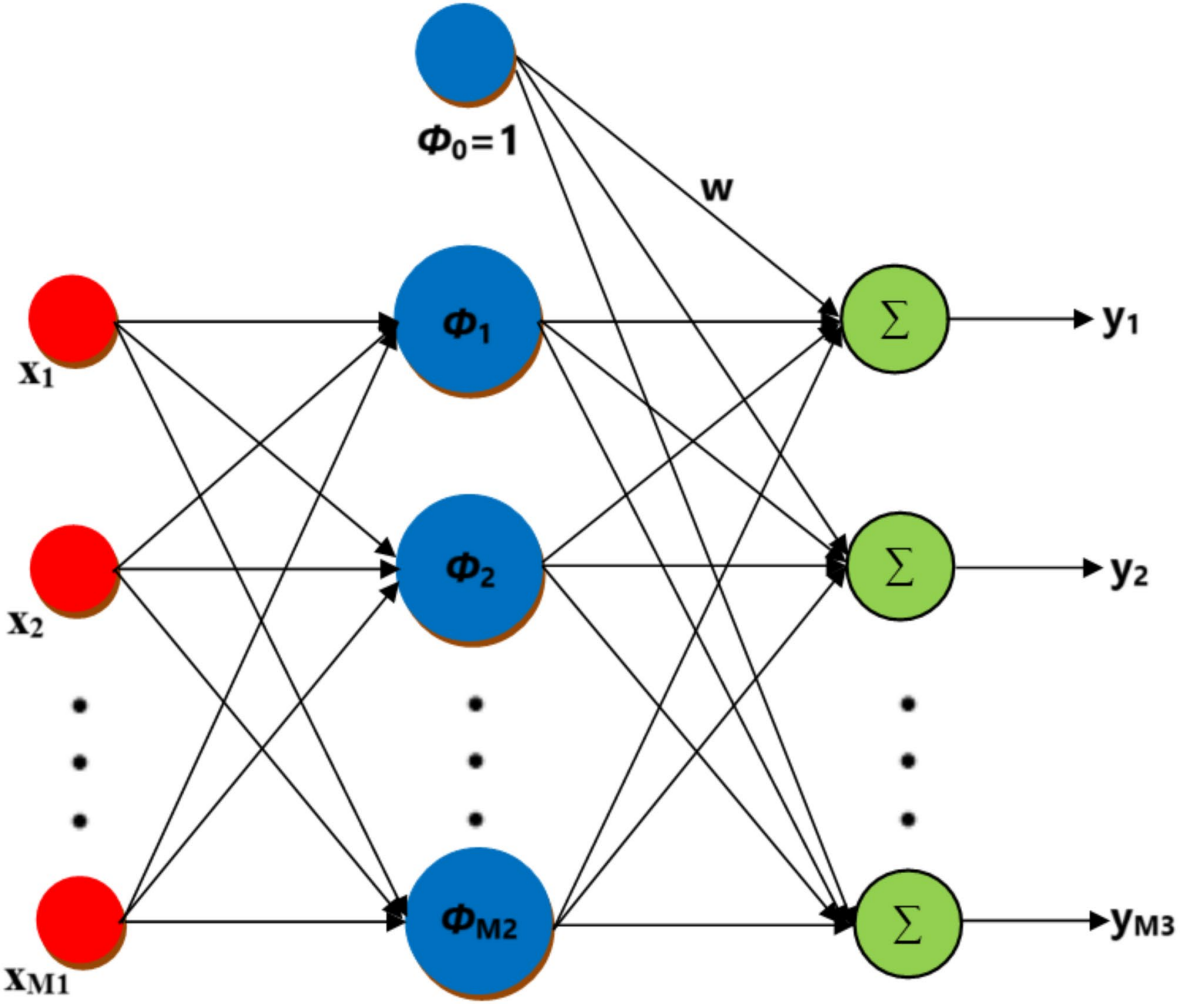
RBFNN architecture.

Broomhead and Lowe developed RBFNN for the first time at the Royal Signals and Radar Establishment^40^. The RBFNN serves as an alternative to multilayer perceptron neural networks and functions as a universal approximator. The RBFNN features a simple architecture and employs the radial basis function as its activation function. It is utilized for function approximation, classification, and time series forecasting. According to Fig. 1, the RBFNN is an M1-M2-M3 feed-forward neural network. M1 nodes are present in the input layer, while M2 and M3 neurons are in the hidden and output layers.

The output bias is denoted by $\:{\phi\:}_{o}\left(x\right)=1$, while $\phi _{i} \left( x \right) = \phi \left( {x - c_{i} } \right)$, $\:{c}_{i}$ represent the *i-*th node center and $\:\phi\:\left(x\right)$ in radial basis function^41,42^. The input is transformed nonlinearly by each node in the hidden layer using the radial basis function as its nonlinear activation function. Radial basis function and neuron parameter combinations are used as inputs and outputs for the RBFNN. By including neurons with the same constant activation function $\:{\phi\:}_{o}=1$ as in the hidden layer, the bias of the output layer is modeled. RBFNN uses a linear optimization method to find the ideal weights for minimizing mean square error. The network’s output is represented as a particular input pattern $\:x$:

1 $$\:{y}_{i}\left(x\right)={\sum\:}_{k=1}^{{M}_{2}}\phi\:\left(||x-{c}_{k}||\right){w}_{ki};\hspace{1em}i=1,\dots\:,{M}_{3}$$

Here, $\:{y}_{i}\left(x\right)$ = RBFNN *i-*th output, $\:{c}_{k}$= *k-*th hidden unit center,$\:{w}_{ki}$= connection weight to the *i-*th output from the *k-*th hidden unit and Euclidean norm represented by $\:||.||$.

RBFNN training is mainly done in two parts. First, the hidden layer’s center is chosen using random sampling or k-mean clustering, and then the model is fitted linearly in the next step.

### Long short-term memory neural network (LSTMNN)

**Fig. 2:**
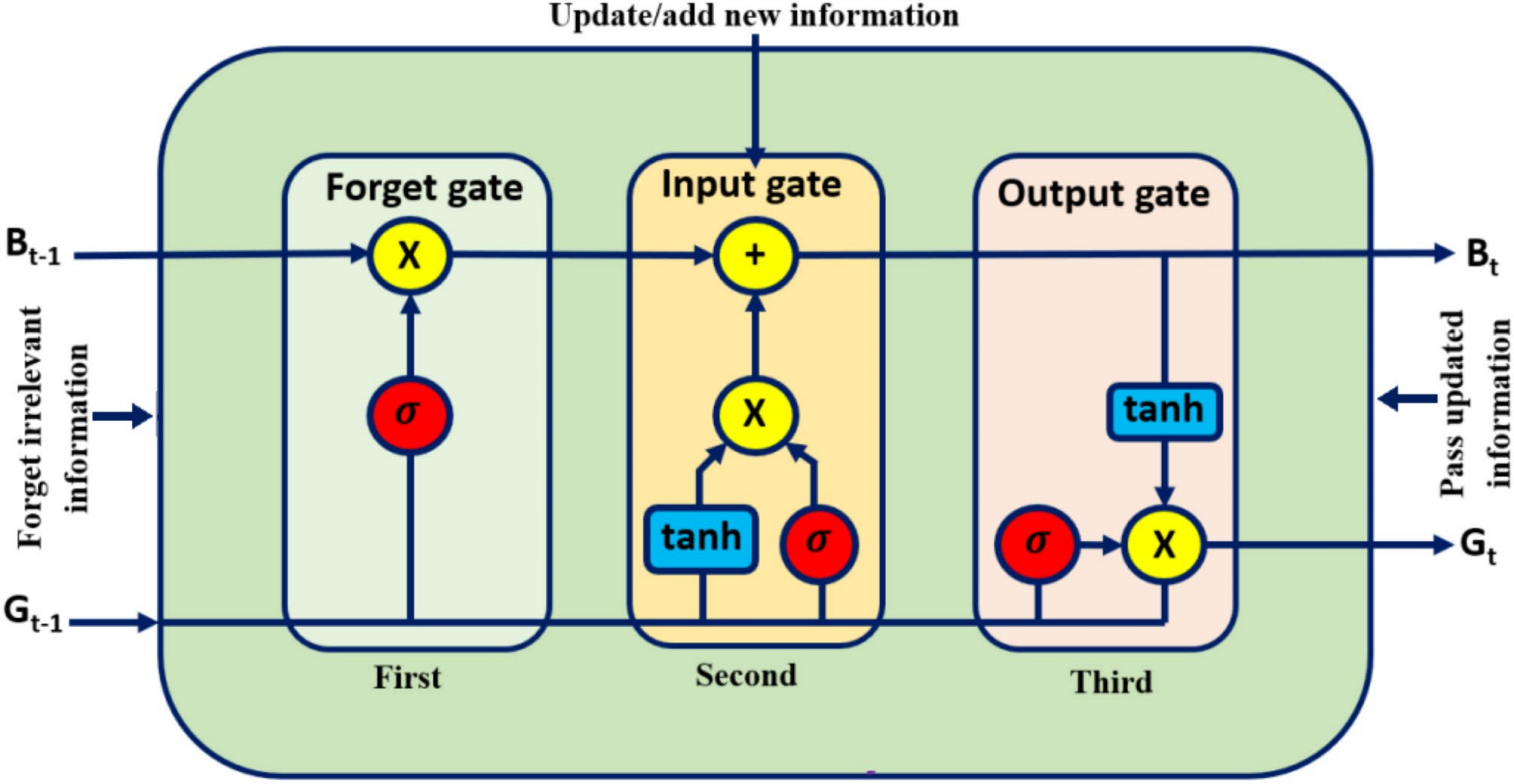
LSTMNN layer.

Recurrent neural networks (RNNs) confront the vanishing gradient problem, which can only be resolved by a specific type of RNN. Hochreiter and Schmidhuber developed the LSTMNN to overcome the difficulty posed by traditional RNN and machine learning algorithms^43,44^. LSTMNN may be implemented by utilizing the Keras package in Python. Let’s pretend you can recall what happened in the last movie scene or the events of the last chapter of your favourite book. Like RNNs, they take in data from the past and use it to understand the present better. Due to the decaying gradient, RNNs suffer from a lack of long-term dependency memory. When building LSTMNNs, long-term reliance problems are particularly avoided. According to Fig. 2, the LSTMNN design is divided into three Sects^45,46^. The first level determines whether the information from the prior time stamp should be retained. The second layer’s cell uses this one as an input when it tries to learn anything new. The cell transmits the updated data from the current time stamp when it reaches the third layer. One single-time step is considered to be one LSTMNN cycle. Each of these three parts makes up one gate of an LSTMNN unit. They regulate the information that enters and exits the LSTMNN cell, also known as the memory cell.

The first of these gates is known as the forget gate, while the other two gates are input and output, respectively. In a conventional feed-forward neural network, each neuron possesses a hidden layer and a present state; hence, each LSTM unit consists of three gates, and a memory cell may be likened to a layer of neurons inside the network. Similar to RNN, LSTMNN also comprises a hidden state, denoted as $\:{G}_{t}$ for the current timestamp and $\:{G}_{t-1}$ for the past timestamp. Furthermore, LSTMNN includes a cell state denoted as $\:{B}_{t}$ for the present time stamp and $\:{B}_{t-1}$ for the prior time stamp. Here, long term memory refers to the visible cell state and short term memory to the hidden state.

#### Forget gate

Whether to maintain or throw away the data from the previous time step is the first choice made in a cell of the LSTMNN. The forget gate formula is displayed below.

2 $$\:{f}_{t}=\sigma\:\left({U}_{f}\times\:{x}_{t}+{w}_{f}\times\:{G}_{t-1}\right)$$

Here, $\:{U}_{f}$= weight connected to the input, $\:{x}_{t}$= current time stamp input, $\:{w}_{f}$= weight matrix related to hidden state, $\:{G}_{t-1}$= previous time stamp hidden state.

A sigmoid function is then used to make $\:{f}_{t}$ a positive integer (between 0 and 1). The cellular state at the previous time stamp is then multiplied by this $\:{f}_{t}$, as follows;

3 $$\:{f}_{t}\times\:{B}_{t-1}=0\hspace{1em}if\hspace{0.33em}{f}_{t}=0\:\left(\text{F}\text{o}\text{r}\text{g}\text{e}\text{t}\:\text{e}\text{v}\text{e}\text{r}\text{y}\text{t}\text{h}\text{i}\text{n}\text{g}\right)$$

4 $$\:{f}_{t}\times\:\:{B}_{t-1}={B}_{t-1}\hspace{1em}if\hspace{0.33em}{f}_{t}=1\:\left(\text{F}\text{o}\text{r}\text{g}\text{e}\text{t}\:\text{n}\text{o}\text{t}\text{h}\text{i}\text{n}\text{g}\right)$$

#### Input gate

The relevance of new data brought in by the input is evaluated using the input gate. The following equation describes the input gate.

5 $$\:{i}_{t}=\sigma\:\left({U}_{i}\times\:{x}_{t}+{w}_{i}\times\:{G}_{t-1}\right)$$

Here, $\:{U}_{i}$= input weight matrix, $\:{w}_{i}$= input weight matrix connected with the hidden state, $\:{x}_{t}$= input at the current time stamp, $\:{G}_{t-1}$= hidden state at the previous time stamp. The sigmoid function ensures that the output lies between 0 and 1.

#### New information

A hidden state at a prior timestamp is determined by the most recent information that must be provided to the cell state at $\:t-1\:$and the input $\:x$ at timestamp $\:t$. As $\:tanh$ is an activation function, the value of new information will vary between − 1 and 1. For negative $\:{N}_{t}$, the information is eliminated from the cell state; otherwise, it is incorporated into the cell state at the present time stamp.

6 $$\:{B}_{t}={f}_{t}\times\:{B}_{t-1}+{i}_{t}\times\:{N}_{t}$$

#### Output gate

The equation for the output gate is provided as,

7 $$\:{O}_{t}=\sigma\:\left({U}_{o}\times\:{x}_{t}+{w}_{o}\times\:{G}_{t-1}\right)$$

This sigmoid function guarantees that the result will be between zero and one. The current cell state $\:tanh$ and $\:{O}_{t}$ are utilized to infer the hidden state.

8 $$\:{G}_{t}={O}_{t}\times\:{tan}h({B}_{t})$$

It turns out that the hidden state is influenced by both the present output and the long-term memory ($\:{B}_{t}$). To obtain the current timestamp output, activate the hidden state, $\:{G}_{t}$, using $\:SoftMax$ activation.

9 $$\:Output=SoftMax\left({G}_{t}\right)$$

### Modular neural network (MNN)

**Fig. 3:**
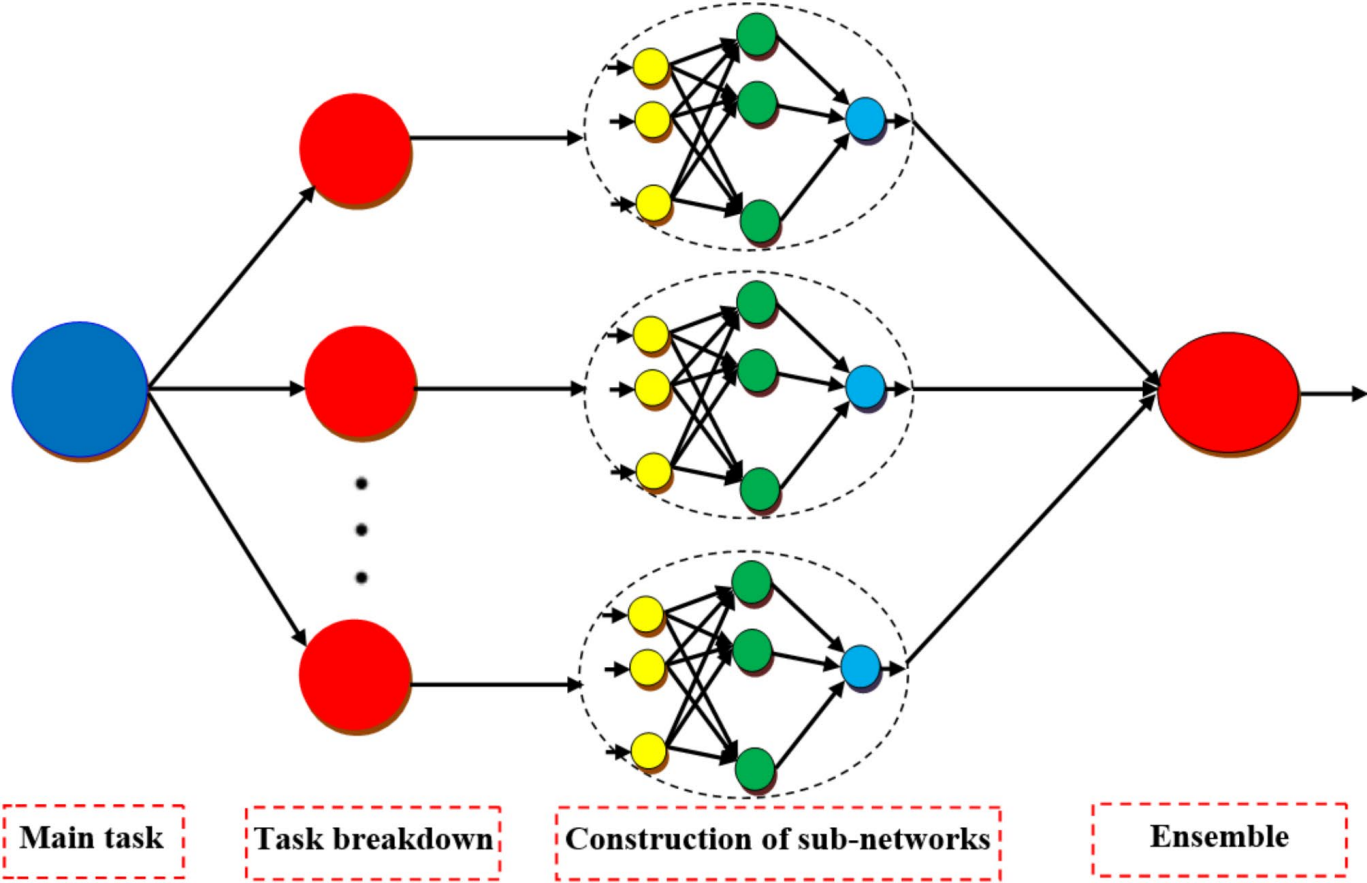
Architecture of MNN.

Often, a single ANN cannot deliver satisfactory performance per the issue and needs. As a result, many ANNs are required to address the same problem. MNNs are used to do this, as presented in Fig. 3^47^.

MNN uses several ANNs as modules to address the entire situation. Each ANN stands for a module and is responsible for resolving a particular aspect of the issue. In this way, action is conducted with the assistance of all the ANNs. The problem must now be broken down into its many parts. Here, each ANN or module is provided input specific to its function. The numerous modules each address a portion of the issue on their own. These do their computations. An integrator obtains the computed outcomes. The integrator’s role is to consolidate the many individual responses from several ANNs and generate a unified output for the system. The complete system may include two components that function in a master-slave configuration. The initial component comprises an integrator that decomposes the issue into its fundamental parts and assigns them to the following modules. This then aggregates the responses from the several modules and computes the system’s ultimate output. The modules represent distinct components; each module is an ANN.

### Transformer model (TM)

**Fig. 4:**
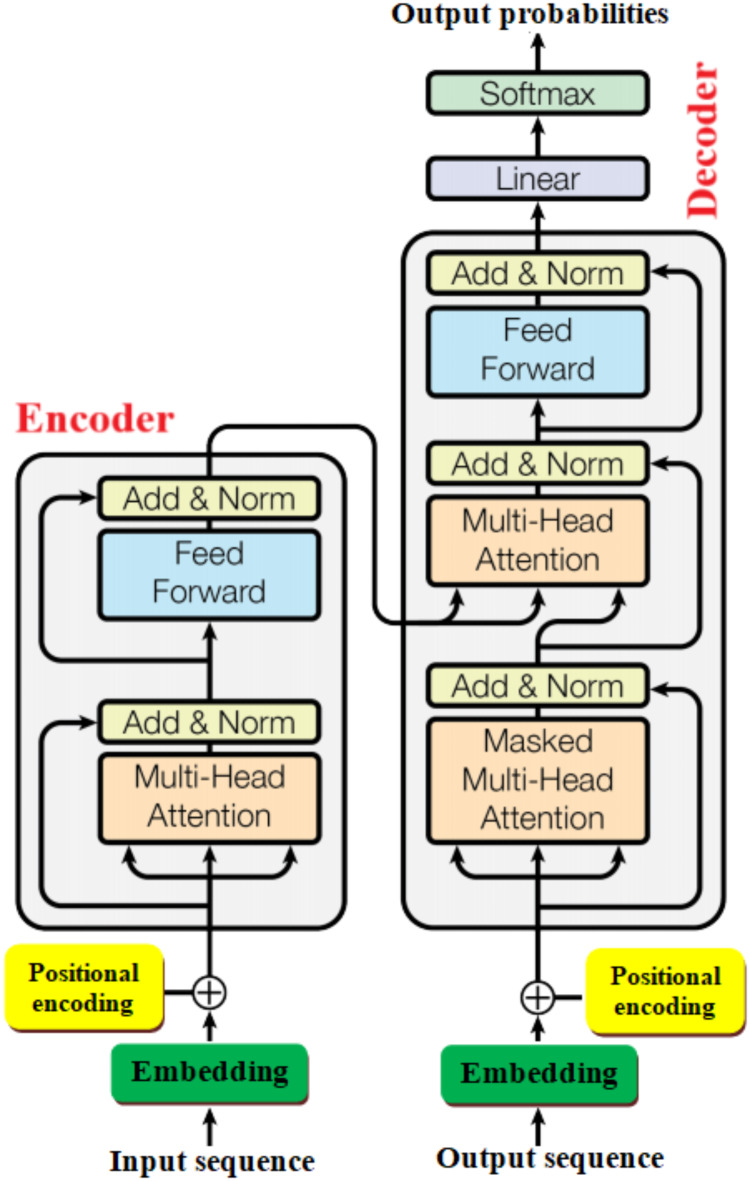
Transformer model^48^ (Freely accessible).

Contextualized representations of the input tokens are created by the encoder as its main purpose. The functionality of the transformer encoder is that it records the input context for each token in relation to the complete input sequence. Transformer developed by eight scientists working at Google^48^ as shown in Fig. 4. It is a deep learning model for working with sequential data like text, speech, or time-series data. It is based on attention mechanism. It is the first completely attention-based sequence transduction model, replacing the recurrent layers commonly seen in encoder-decoder designs with multi-headed self-attention. The transformer may be learned much faster for translation applications than systems based on convolution or recurrent layers. The key components of this model are as following:

- Multi-head attention: Enables various input sequence segments to be handled by the model.
- Positional encoding: Add details regarding the sequence’s order.
- Feed forward network: Following attention, a completely connected network was applied.
- Layer normalization and residual connections: Assist in bringing the training process under control.

### DGSR prediction

**Fig. 5:**
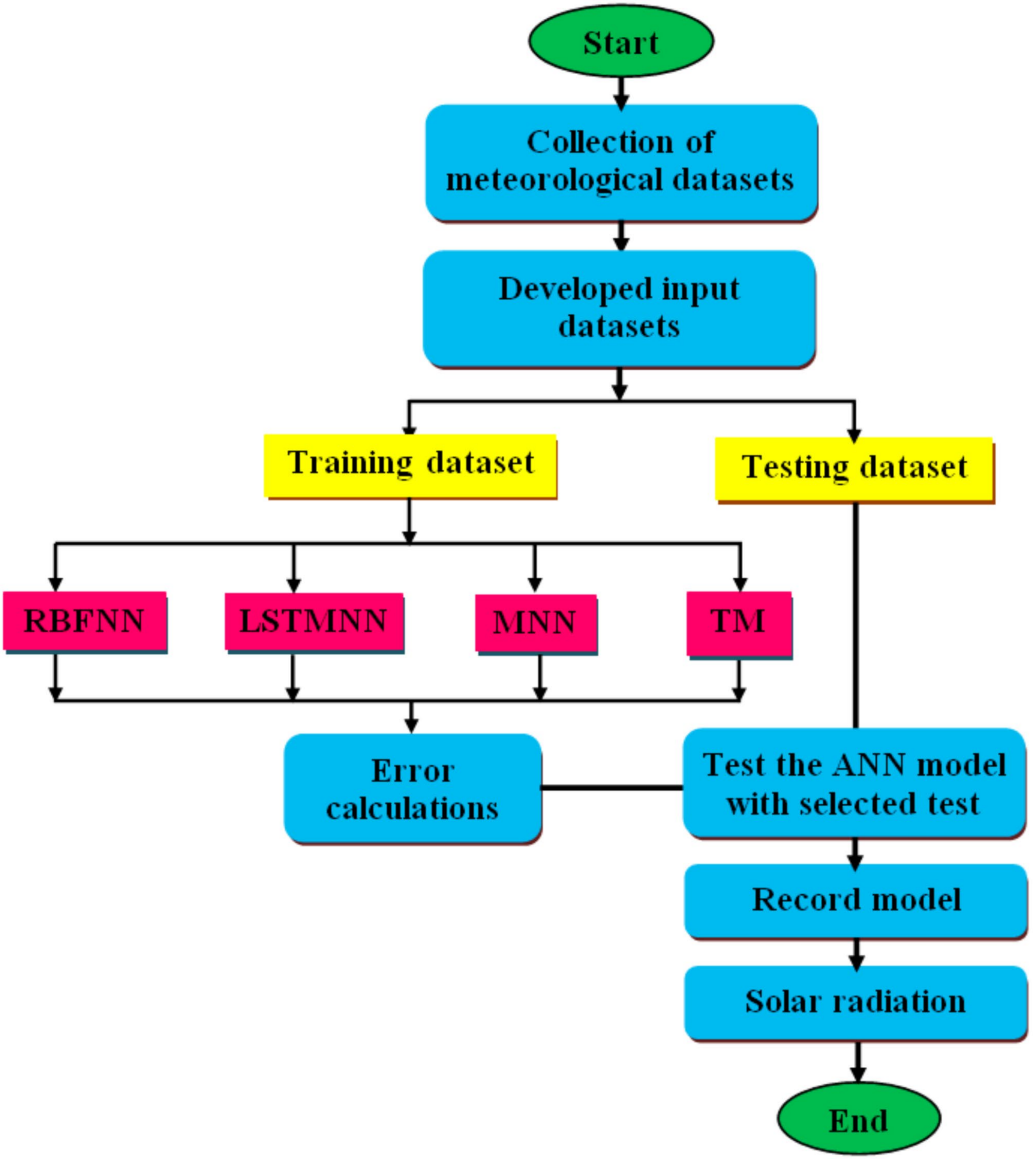
DGSR prediction model based on different ANN models.

For the prediction of DGSR, different ANN models are developed with varying combinations of input, which are shown in Table 3. All ANN models are tested using 172 data samples after training with 680 data samples for DGSR prediction. In Fig. 5, the DGSR prediction based on several models is displayed. Equation 10 to 19 shows, Mean Squared Error (MSE), Root Mean Squared Error (RMSE), Mean bias error (MBE), Mean Absolute Error (MAE), Mean Absolute Relative Error (MARE), Mean Squared Relative Error (MSRE), Root Mean Squared Relative Error (RMSRE), Root Mean Squared Percentage Error (RMSPE), Coefficient of Determination (R^2^) and correlation regression (R) are used to analyze prediction error and determined as^49–55^:

10 $$\:MSE=\frac{1}{n}{\sum\:}_{q=1}^{n}({T}_{q}-O{A}_{q}{)}^{2}$$

11 $$\:RMSE=\sqrt{\frac{1}{n}{\sum\:}_{q=1}^{n}({T}_{q}-O{A}_{q}{)}^{2}}$$

12 $$\:\:\text{M}\text{B}\text{E}=\frac{1}{n}\sum\:_{q=1}^{n}({T}_{q}-O{A}_{q})$$

13 $$\:\text{M}\text{A}\text{E}=\:\frac{1}{n}\sum\:_{q=1}^{n}\left|{T}_{q}-O{A}_{q}\right|$$

14 $$\:\:\text{M}\text{A}\text{R}\text{E}=\frac{1}{n}\sum\:_{q=1}^{n}\frac{\left|{T}_{q}-O{A}_{q}\right|}{\left|{T}_{q}\right|}$$

15 $$\:\text{M}\text{S}\text{R}\text{E}=\frac{1}{n}{\sum\:}_{q=1}^{n}{\left(\frac{{T}_{q}-O{A}_{q}}{{T}_{q}}\right)}^{2}$$

16 $$\:\text{R}\text{M}\text{S}\text{R}\text{E}=\sqrt{\frac{1}{n}{\sum\:}_{q=1}^{n}{\left(\frac{{T}_{q}-O{A}_{q}}{{T}_{q}}\right)}^{2}}$$

17 $$\:\text{R}\text{M}\text{S}\text{P}\text{E}=\sqrt{\frac{1}{n}{\sum\:}_{q=1}^{n}{\left(\frac{{T}_{q}-O{A}_{q}}{{T}_{q}}\:\times\:100\right)}^{2}}$$

18 $$\:\text{R}2\hspace{0.17em}=\hspace{0.17em}1-\frac{\sum\:_{i=1}^{n}{\left({T}_{q}-O{A}_{q}\right)}^{2}}{\sum\:_{i=1}^{n}{(T}_{q}-\overline{{T}_{q}})}$$

19 $$\:\text{R}=\frac{\left({T}_{q}-\:\overline{{T}_{q}}\right)\left(O{A}_{q}-\overline{O{A}_{q}}\right)}{\sqrt{\sum\:_{i=1}^{n}{\left({T}_{q}-\:\overline{{T}_{q}}\right)}^{2}}\sqrt{\sum\:_{i=1}^{n}{\left(O{A}_{q}-\:\overline{O{A}_{q}}\right)}^{2}}}$$

Here, $\:n$ = data set samples, $\:O{A}_{q}$= predicted solar radiation, $\:{T}_{q}$=measured solar radiation, $\:\overline{{T}_{q}}=$ mean value of measured solar radiation,$\:\:\:\overline{O{A}_{q}}$=mean value of predicted solar radiation.

MSE measures the average squared difference between measured and predicted solar radiation values. Low MSE indicates better performance. The RMSE estimate predicts the error in the same unit as the measured solar radiation. RMSE penalizes large errors more, so it is useful for high-variance datasets. MBE indicates bias in the solar radiation prediction model. Positive MBEs indicate overestimation, and negatives indicate underestimation. MAE measures the absolute deviation of predicted solar radiation from the measured solar radiation value. It is less sensitive to large errors in comparison to RMSE. MARE is similar to the mean absolute percentage error, but it does not include a percentage factor. When the measured value of solar radiation varies widely, we use it to analyze the model. MSRE signifies the impact of relative errors on model accuracy. RMSRE interprets errors in relative terms. RMSPE indicates the relative magnitude of errors in the solar radiation prediction model. R^2^ signifies variance in solar radiation. Its value close to 1 indicates strong prediction ability, while its values near to 0 indicate poor performance. R measures the linear relation’s direction and strength between predicted and measured solar radiation values. An R close to 1 indicates a strong positive correlation.

**Table 3:** Input variable of different models.

ANN models	Input variables				
	WS	RH	T	T_max_	T_min_
LSTMNN-1	✓	✓	✓	✓	✓
LSTMNN-2	×	✓	✓	✓	✓
LSTMNN-3	✓	×	✓	✓	✓
LSTMNN-4	×	×	✓	✓	✓
MNN-1	✓	✓	✓	✓	✓
MNN-2	×	✓	✓	✓	✓
MNN-3	✓	×	✓	✓	✓
MNN-4	×	×	✓	✓	✓
RBFNN-1	✓	✓	✓	✓	✓
RBFNN-2	×	✓	✓	✓	✓
RBFNN-3	✓	×	✓	✓	✓
RBFNN-4	×	×	✓	✓	✓
TM-1	✓	✓	✓	✓	✓
TM-2	×	✓	✓	✓	✓
TM-3	✓	×	✓	✓	✓
TM-4	×	×	✓	✓	✓

## Data collection

**Fig. 6:**
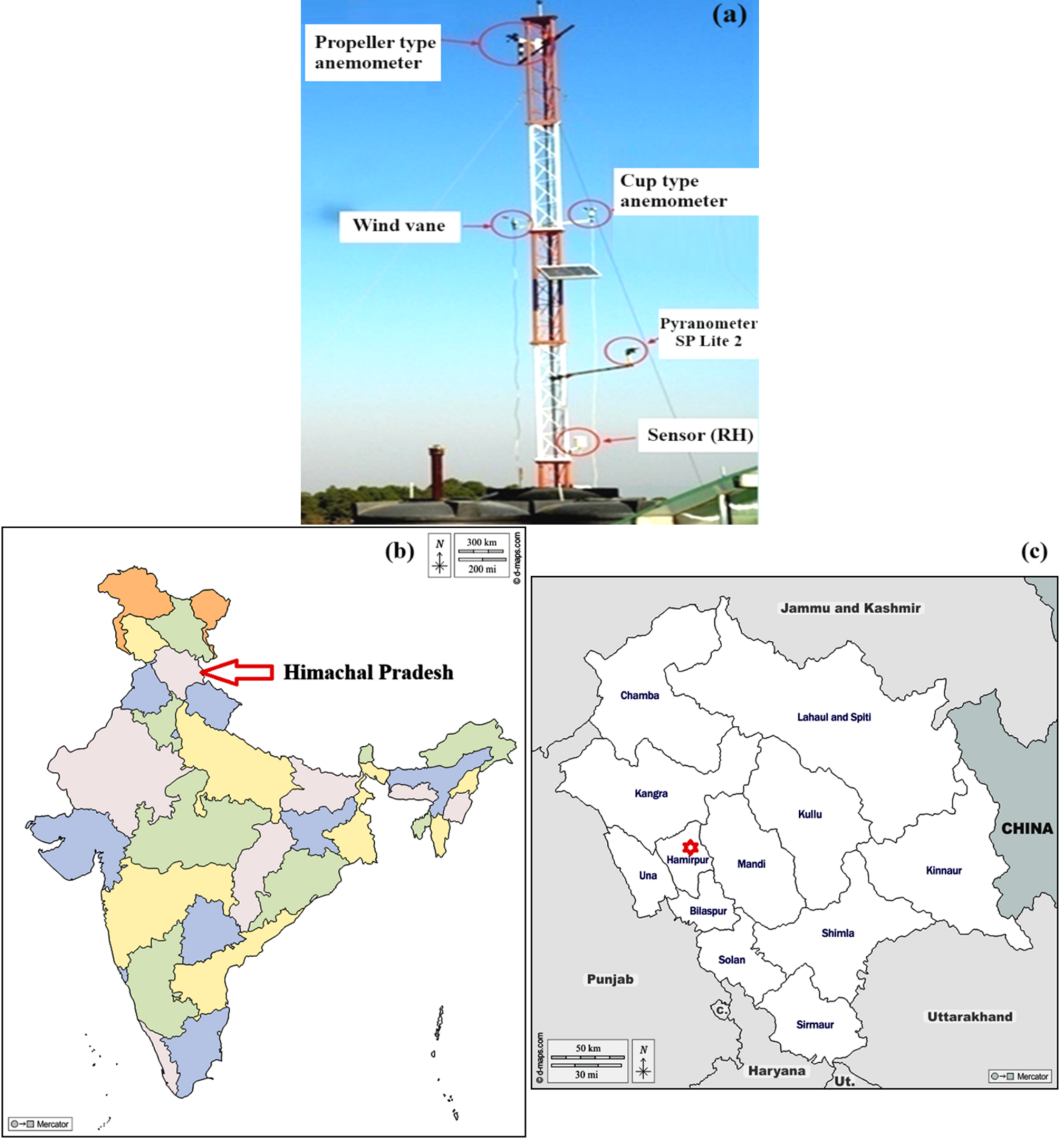
(**a**) Automatic weather station, (**b**) Map of India showing Himachal Pradesh, (**c**) Hamirpur district location in Himachal Pradesh (**b**,** c** are downloaded from https://d-maps.com).

The automatic weather station installed at the Centre for Energy Studies, National Institute of Technology Hamirpur India provided the measure values for the meteorological variables (T, DGSR, RH, and WS), as presented in Fig. 6a^56^. Based on the map of India (Fig. 6b), the experimental set-up is located at 31.68 North, longitude 78.52 East by an altitude of 785 m in Himachal Pradesh (Fig. 6c). The 852 real data measured values from 1 January 2012 to 1 May 2014 are shown in the 10-minute daily average value of RH, T_min_, T_max_, WS, T, and DGSR. Table 4 displays the precise specifications and instrument accuracy. A comprehensive overview of the collected datasets (WS, RH, T, T_max_, T_min_, and DGSR) is presented in Table S1 (Supplementary Material).

**Table 4:** Measuring instruments specifications.

	Anemometer		Sensor			
	Handheld KM-909	Propeller type RMY-5,010,310 L	Radiation SP LITE-2	Humidity *P*-41,342	Temperature *P*-41,342	Pressure *P* 61,302 V
Range	0–30 m/s	0–100 m/s	400–1100 nm	0-100%RH	− 50 to +50 °C	500–1100 hPa
Resolution	0.01 m/s	0.001 m/s	0.001 W/m^2^	1%	0.1 °C	0.01 hPa
Accuracy	± 3% m/s	± 0.3 m/s	± 0.5 mV/W/m^2^	± 1%RH	± 0.3 °C	± 0.3 hPa
Operating temperature	0 °C to 50 °C	− 50 °C to + 50 °C	− 30 °C to + 70 °C	− 50 °C to + 50 °C	− 50 °C to + 50 °C	− 40 °C to + 60 °C
Output	Voltage	AC voltage	Voltage	Voltage	Voltage	Voltage

The database’s descriptive statistics are displayed in Table 5, which primarily contains mean, median, mode, standard deviation, standard error, sample variance, confidence level, and maximum/minimum values. As can be shown from Table 3 data analysis, the highest DGSR was 0.6116 kWh/m^2^/day. The average DGSR was determined to be 0.179 kWh/m^2^/day based on the data gathered, with a standard deviation of 0.1482 kWh/m^2^/day, a confidence level of 0.01 and a standard error of 0.0051 kWh/m^2^/day.

**Table 5:** Descriptive analysis of input and output variables.

	Input variables					Output variable
	WS	RH	T	T_max_	T_min_	Solar radiation
Data set	852	852	852	852	852	852
Mean	2.0478	62.5625	20.8789	24.7234	17.4510	0.1790
Median	1.9460	61.9943	21.8240	25.1900	18.0350	0.1552
Mode	1.5168	100.0000	23.6569	19.6100	22.9600	0.0015
SD	0.5777	22.7882	6.3062	6.5953	6.0725	0.1482
SE	0.0198	0.7807	0.2160	0.2260	0.2080	0.0051
SV	0.3337	519.3026	39.7687	43.4985	36.8750	0.0220
MAD	0.4448	19.2701	5.2730	5.4245	5.1911	0.1258
Minimum	0.7691	11.7192	4.0045	5.8410	2.5840	0.000006
Maximum	4.4662	100.0000	37.8407	40.4600	34.7500	0.6116
Kurtosis	1.5184	− 0.9744	− 0.6245	− 0.5132	− 0.8486	− 0.6326
Skewness	1.0208	− 0.0942	− 0.1017	− 0.1545	− 0.1051	0.5942
Range	3.6971	88.2808	33.8362	34.6190	32.1660	0.6116
CL (95.0%)	0.0388	1.5323	0.4240	0.4435	0.4083	0.0100

*SD* standard deviation, *SE* standard error, *MAD* mean absolute deviation, *SV* sample variance, *CL* confidence level.

**Fig. 7:**
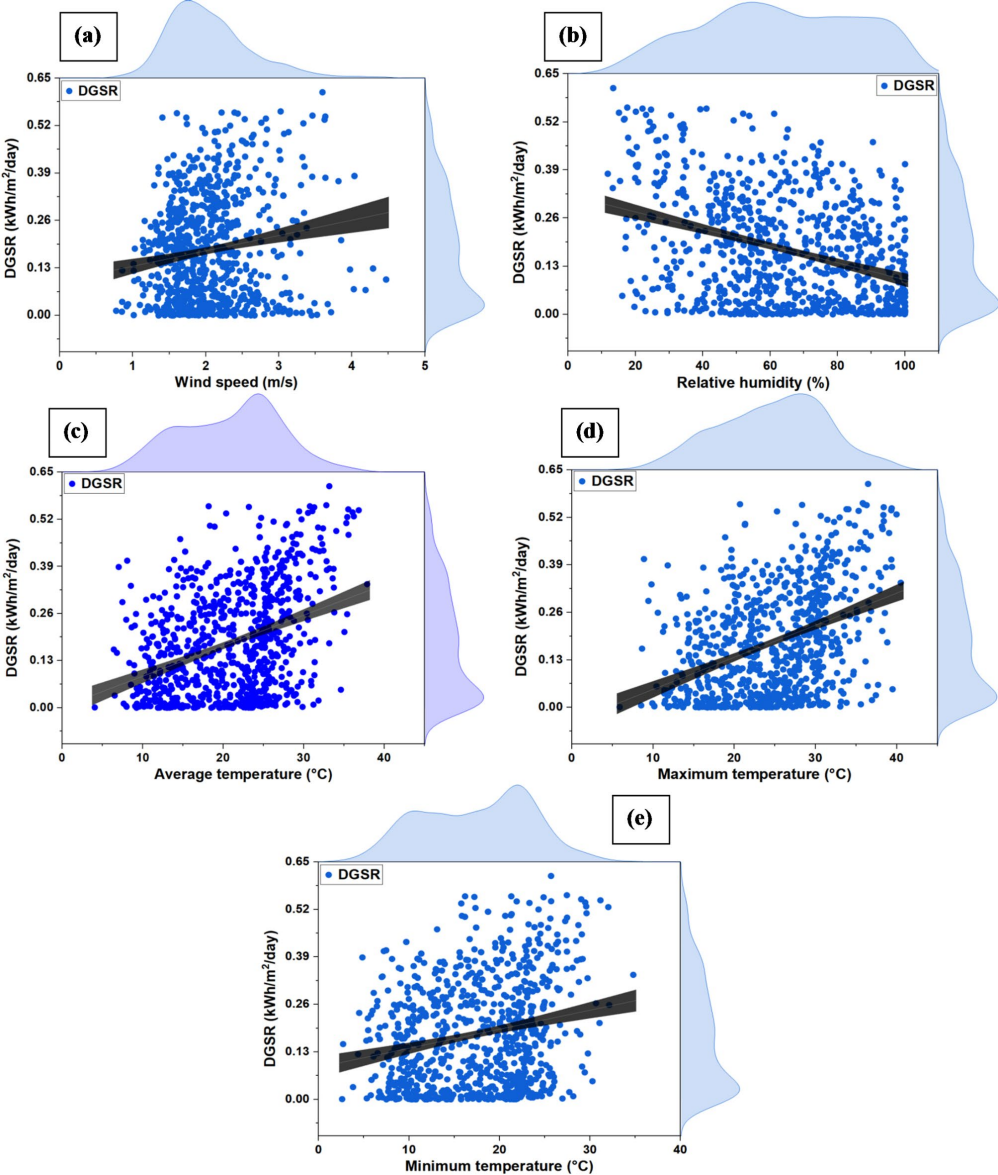
Distribution DGSR for various input variables, (**a**) WS, (**b**) RH, (**c**) T, (**d**) T_max_, (**e**) T_min_.

**Fig. 8:**
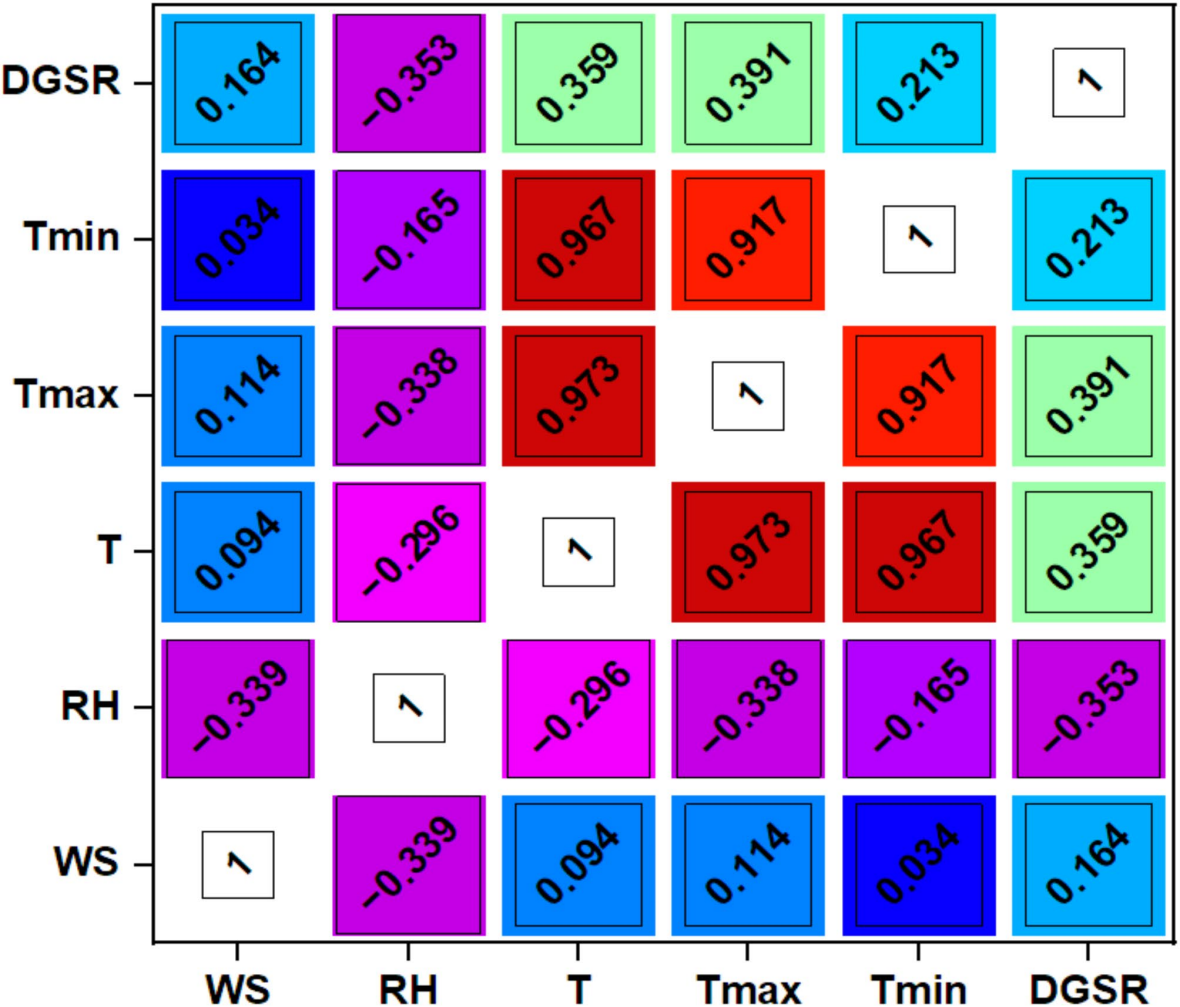
Pearson correlation plots between inputs and target variables.

Figure 7 visualizes the values of input variables and the corresponding distribution of DGSR. The right-hand axis in each image in Fig. 7 represents the visible distribution density of the output variable (DGSR). In contrast, the upper-hand axis corresponds to the associated input variables. Figure 7 shows that the distribution range of the output variable (DGSR), which corresponds to the values of each input variable, is discrete and relatively wide. As a result, before algorithm training, the statistical correlation between the data must be analyzed. The Pearson’s correlation plot is shown in Fig. 8 showing RH has less value of correlation with DGSR and T_max_ has more correlation value. Moreover the correlations between the input variables (WS, RH, T, T_max_, and T_min_) with output target (DGSR) were within the 0.5 threshold.

The findings show that the validity of the datasets has been confirmed, allowing them to be used for subsequent algorithmic model analysis.

## Results and discussions

### Performance evaluation of ANN models

**Fig. 9:**
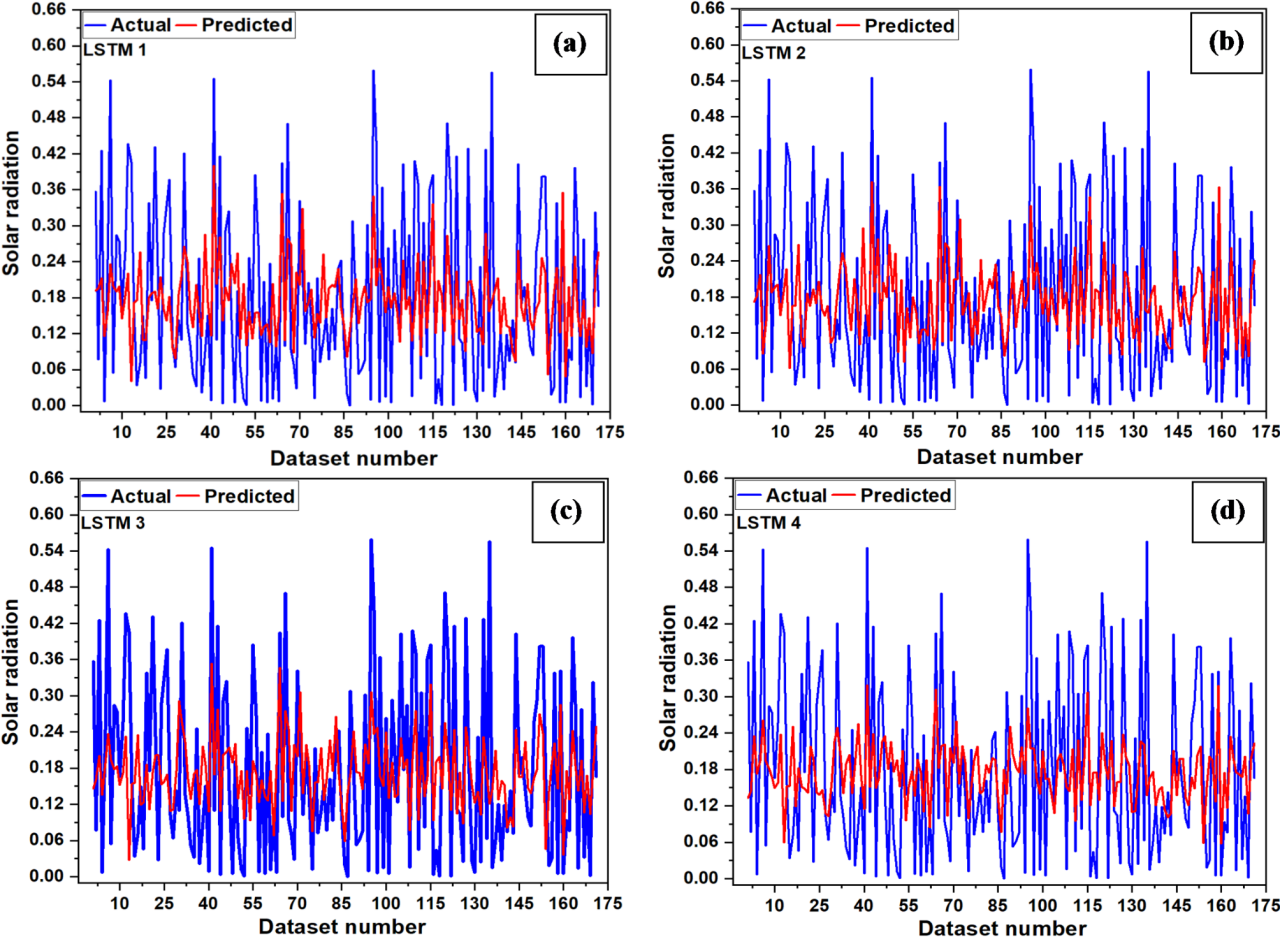
Testing plots (**a**) LSTMNN-1, (**b**) LSTMNN-2, (**c**) LSTMNN-3, (**d**) LSTMNN-4.

The variables used to train ANN models are listed in Table 3, and the corresponding error analysis for various sets of input variables are shown in Table 6. The testing plot of LSTMNN is shown in Fig. 9. It was found that MSE and MBE vary from 0.016 to 0.018 and − 0.006 to 0.108, respectively. The recorded lowest RMSE (0.126) and highest R (0.290) show RH, T, T_max_ and T_min_ are essential variables for solar radiation prediction using the LSTMNN-2 model. Meanwhile, with the highest recorded RMSE of 0.137 along with least R of 0.15, T, T_max_ and T_min_ are the least important variables for solar radiation prediction using the LSTMNN-4 model.

**Fig. 10:**
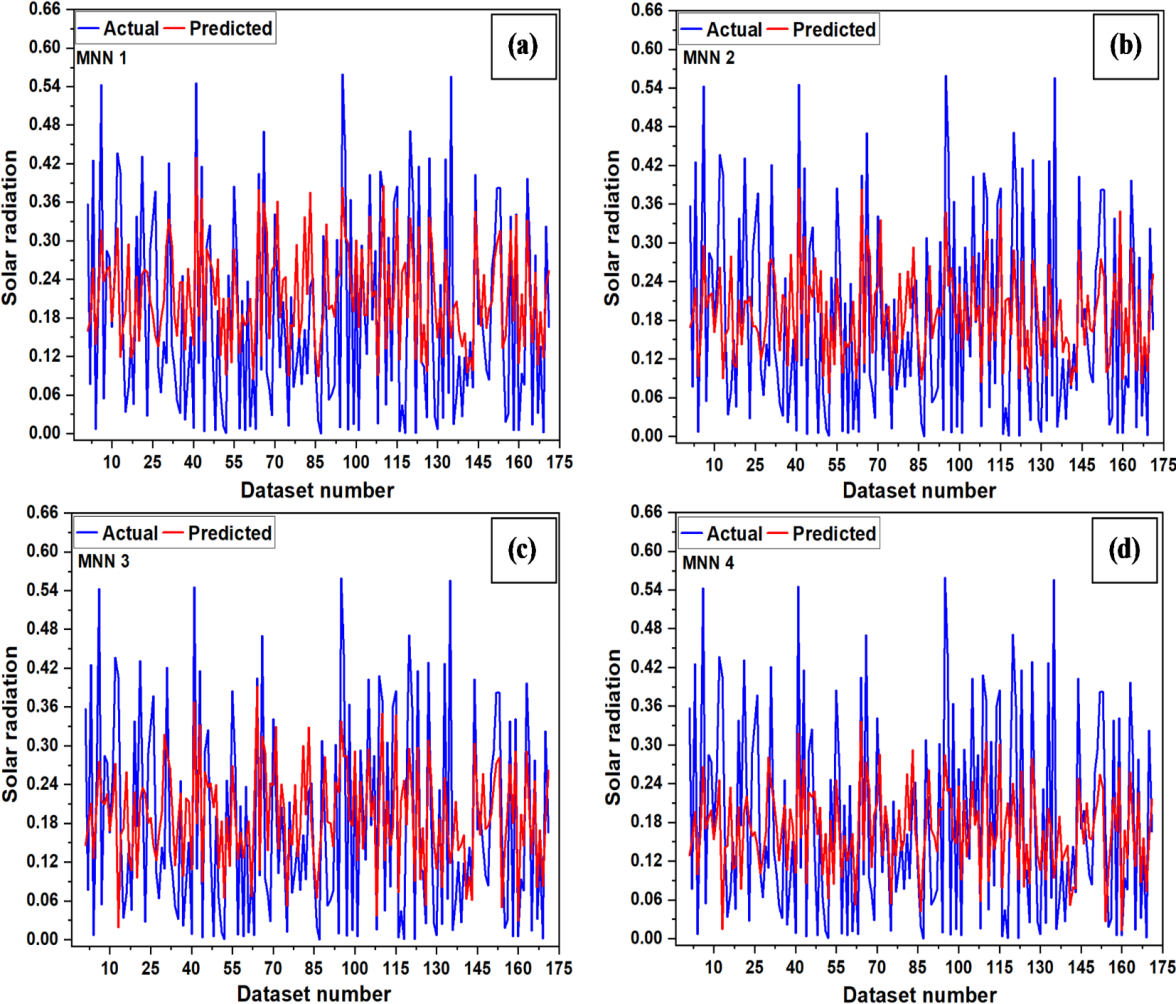
Testing plots (**a**) MNN-1, (**b**) MNN-2, (**c**) MNN-3, (**d**) MNN-4.

The testing plots of MNN networks are shown in Fig. 10. It was found that MSE and MBE vary from 0.014 to 0.015 and 0.0084 to 0.034, respectively. The lowest RMSE of 0.120 along with a high R value of 0.340 suggests that the RH, T, T_max_ and T_min_ remain the essential variables for solar radiation prediction using the MNN-2 model, while T, T_max_, and T_min_ are the least important variable for solar radiation prediction, as evidenced from the high RMSE of 0.123 and low R value of 0.320 for MNN-4 model.

**Table 6:** Errors of different models.

ANN models	Input variables	MSE	RMSE	MBE	*R*	MAE	MARE	MSRE	RMSRE	RMSPE	*R* ^2^
LSTMNN-1	WS, RH, T, T_max_, T_min_	0.016	0.128	0.108	0.259	**0.10**	7.77	1035.44	32.17	3217.82	0.25
LSTMNN-2	RH, T, T_max_, T_min_	0.016	0.126	− 0.006	0.290	**0.10**	7.07	843.35	29.04	2904.06	0.29
LSTMNN-3	WS, T, T_max,_ T_min_	0.016	0.127	− 0.006	0.270	**0.10**	8.00	1247.03	35.31	3531.34	0.27
LSTMNN-4	T, T_max_, T_min_	0.018	0.137	− 0.005	0.150	0.11	9.73	2058.96	45.37	4537.58	0.15
MNN-1	WS, RH, T, T_max_, T_min_	**0.014**	0.122	0.034	0.330	**0.10**	7.60	1023.23	31.98	3198.80	0.34
MNN-2	RH, T, T_max_, T_min_	**0.014**	**0.120**	0.0084	0.340	**0.10**	7.60	1023.23	31.98	3198.80	0.34
MNN-3	WS, T, T_max,_ T_min_	**0.014**	0.122	0.01	0.330	**0.10**	7.73	1174.08	34.26	3426.49	0.33
MNN-4	T, T_max_, T_min_	0.015	0.123	0.012	0.320	**0.10**	6.99	1010.96	31.79	3179.56	0.32
RBFNN-1	WS, RH, T, T_max_, T_min_	0.019	0.140	0.001	0.119	0.11	9.45	1613.54	40.16	4016.89	0.11
RBFNN-2	RH, T, T_max_, T_min_	0.020	0.144	0.0015	0.060	0.12	11.16	2540.54	50.40	5040.37	0.009
RBFNN-3	WS, T, T_max,_ T_min_	0.020	0.141	**0.0008**	0.100	0.11	10.33	2128.44	46.13	4613.50	0.10
RBFNN-4	T, T_max_, T_min_	0.022	0.150	0.003	0.017	0.12	11.38	2945.25	54.27	5427.02	− 0.017
TM-1	WS, RH, T, T_max_, T_min_	0.550	0.740	− 0.051	0.457	0.58	2.15	157.17	12.53	1253.68	0.43
TM-2	RH, T, T_max_, T_min_	0.546	0.739	0.0254	**0.463**	0.57	2.09	111.93	10.57	1057.99	**0.44**
TM-3	WS, T, T_max,_ T_min_	0.569	0.754	− 0.020	0.440	0.56	1.99	108.04	10.39	1039.43	**0.44**
TM-4	T, T_max_, T_min_	0.564	0.751	− 0.026	0.440	0.58	**1.98**	**106.85**	**10.33**	**1033.71**	**0.44**

Significant values are in bold.

**Fig. 11:**
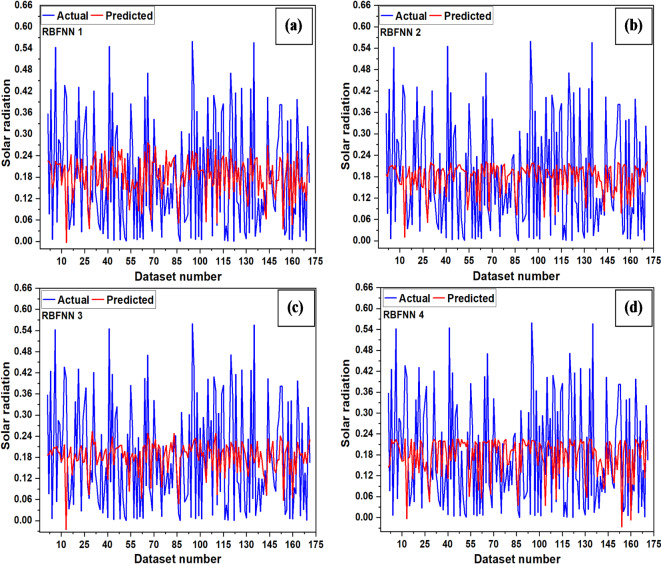
Testing plots (**a**) RBFNN-1, (**b**) RBFNN-2, (**c**) RBFNN-3, (**d**) RBFNN-4.

The testing plots of RBFNN networks are shown in Fig. 11. It was found that for RBFNN models, MSE varies from 0.019 to 0.022, and MBE varies from 0.0008 to 0.003. With the lowest RMSE value of 0.140 along with a high R value (0.119), the WS, RH, T, T_max_ and T_min_ remain essential variables for solar radiation prediction using the RBFNN-1 model. In contrast, the RMSE remains highest (0.150) with a lowest R value (0.017) for the RBFNN-4 model, suggesting that T, T_max_, and T_min_ were the least critical variables in the prediction of solar radiation.

**Fig. 12:**
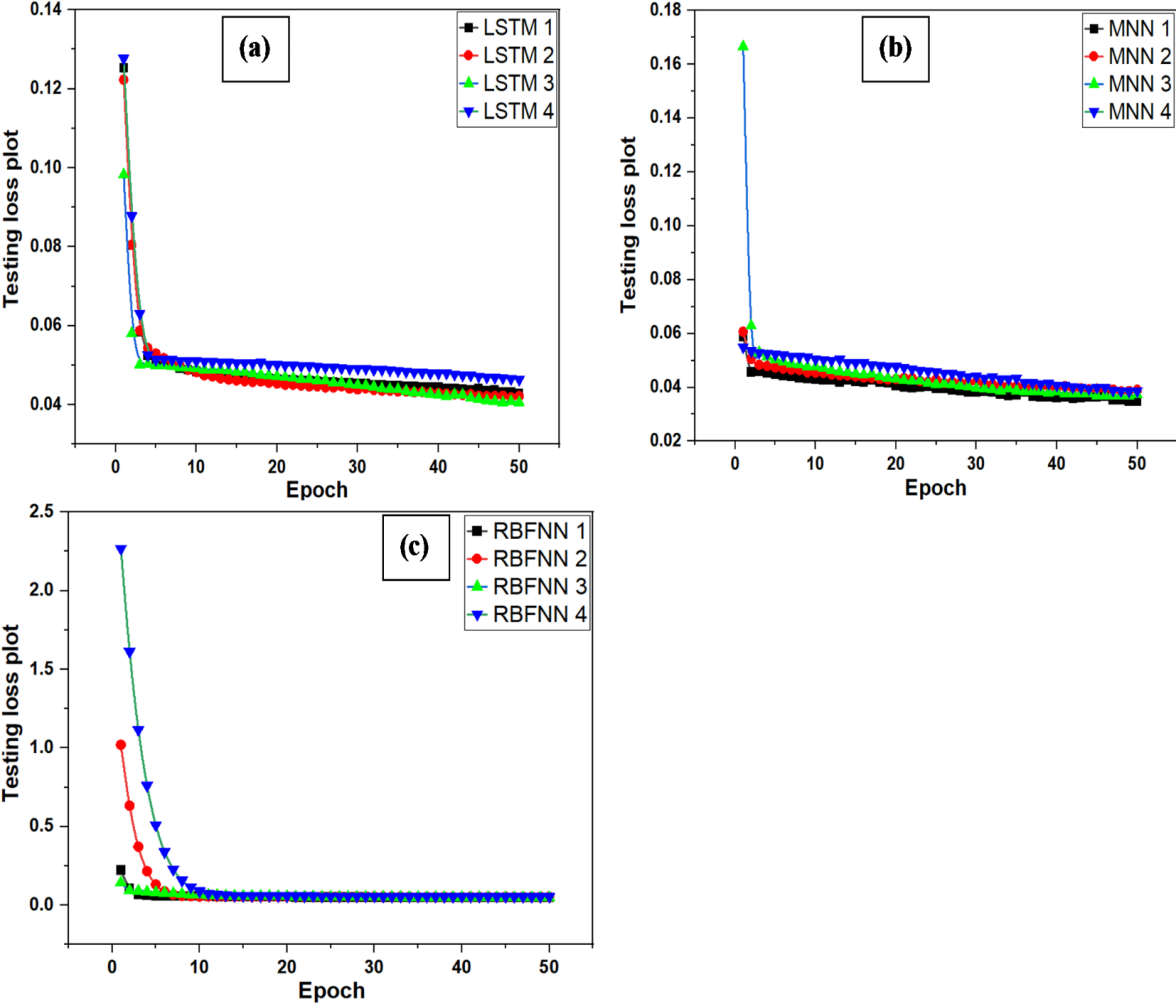
Testing loss plot (**a**) LSTMNN, (**b**) MNN, (**c**) RBFNN.

The training and testing plots of TM networks are shown in Fig. S1 (Supplementary Material). From Table 6, it was found that MSE and MBE vary from 0.546 to 0.569 and − 0.051 to 0.0254, respectively. The recorded lowest RMSE (0.739) and highest R (0.463) show RH, T, T_max_ and T_min_ are essential variables for solar radiation prediction using the TM-2 model. Meanwhile, with the highest recorded RMSE of 0.754 along with least R value of 0.440, WS, T, T_max_ and T_min_ are the least important variables for solar radiation prediction using TM-3 model. The R^2^ value of RBFNN-4 is negative, indicating that the model fits worse than a simple mean prediction of solar radiation. The minimum MSE is observed for MNN-1, MNN-2, and MNN-3. MNN-2 demonstrates the lowest RMSE, whereas RBFNN-3 exhibits a minimal MBE. The R-value of the TM-2 is higher compared to other models. Figure 12 displays the testing loss plots for LSTMNN (Fig. 12a), MNN (Fig. 12b), and RBFNN (Fig. 12c), while for TM is shown in Fig. S2 (Supplementary Material). These plots are developed in google collab by using import matplotlib.pyplot as plt. The testing loss is a criterion employed to see performance of model with epochs which is complete cycle of training and testing. The testing loss graphs demonstrate that the error decreases as the number of epochs increases. It is used to evaluate effective learning of model and generalization capability of model on unseen data. A reduced MSE signifies that the model’s predictions are more accurate than the actual data. Reduced MSE and RMSE signify a more accurate model for projecting solar energy levels about solar radiation prediction with superior predictive capability.

A positive MBE indicates overestimation, whereas a negative MBE signifies a tendency to underestimate. MARE is a valuable metric for evaluating model performance across various datasets or sizes. A reduced MARE in solar radiation forecasting signifies a more reliable and consistent model, particularly when the data exhibits varying magnitudes. Higher R values (closer to 1) indicate that the forecasting model effectively captures the trends and variability in solar radiation. The error analysis for all models is assessed using MSE, RMSE, MBE, MARE, and R values, as shown in Table 6. The MARE for LSTMNN, MNN, RBFNN, and TM ranges from 7.07 to 9.73, 6.99 to 7.73, 9.45 to 11.38, and 1.98 to 2.15, respectively, indicating that TM exhibits the lowest MARE value. Utilizing T, T_max_, and T_min_ as inputs for MNN and TM results in a lower MARE, however it yields a higher MARE for the LSTMNN and RBFNN models. Employing inputs WS, RH, T, T_max_, and T_min_ to MNN, RBFNN, and TM yields a higher MARE.

### Comparison of conventional methods for DGSR prediction

Table 7 displays the standard techniques recommended by different authors^57–61^. The constant parameters a and b are determined by employing least square regression and least square fitting planes. The RMSE, MSE, and MBE for conventional approaches range from 0.26 to 2.87, 0.067 to 8.236, and − 2.85 to 3.52 × 10^− 4^, respectively. The RMSE, MSE, and MBE for ANN models (Table 6) range from 0.120 to 0.754, 0.014 to 0.569, and − 0.051 to 0.108, respectively.

**Table 7:** Error evaluation of various conventional methods.

Conventional methods		Calculated parameters	RMSE	MSE	MBE
Reference	Equations				
[58]	$\:DGSR=a\sqrt{\varDelta\:T}DER$	a = 0.3717	0.37	0.137	− 4.3 × 10^–17^
[59]	$\:DGSR=a(1+1{0}^{-5}\times\:h)\sqrt{\varDelta\:T}DER$	a = 0.3717	0.30	0.090	− 4.3 × 10^–17^
[60]	$\:DGSR=\left(a\sqrt{\varDelta\:T+b}\right)DER$	a = 0.2292 b= − 0.3452	0.26	0.067	3.52 × 10^− 4^
[60]	$\:DGSR=a\sqrt{\varDelta\:T}DER+b$	a = 0.2234 b= − 3.3106	2.87	8.236	− 2.85
[61]	$\:DGSR=\left(a{ln}\varDelta\:T+b\right)DER$	a = 0.2347 b= − 1.9607	2.35	5.522	− 2.33

## Conclusions

This study predicts daily global solar radiation utilizing sixteen models (LSTMNN-1, LSTMNN-2, LSTMNN-3, LSTMNN-4, RBFNN-1, RBFNN-2, RBFNN-3, RBFNN-4, MNN-1, MNN-2, MNN-3, MNN-4, TM-1, TM-2, TM-3, TM-4) including numerous stochastic variables. The wind speed (WS), lowest temperature (T_min_), relative humidity (RH), highest temperature (T_max_), and average temperature (T) are the input variables for RBFNN-1, LSTMNN-1, MNN-1 and TM-1. RBFNN-2, LSTMNN-2, MNN-2 and TM-2 models utilized RH, T, T_max_, and T_min_ as inputs. For RBFNN-3, LSTMNN-3, MNN-3 and TM-3 the inputs WS, T, T_max_, and T_min_ are utilized. RBFNN-4, LSTMNN-4, MNN-4 and TM-4 employ the inputs T, T_max_, and T_min_. The mean absolute relative error for RBFNN-1, RBFNN-2, RBFNN-3, RBFNN-4, LSTMNN-1, LSTMNN-2, LSTMNN-3, LSTMNN-4, MNN-1, MNN-2, MNN-3, MNN-4, TM-1, TM-2, TM -3, TM-4 are 9.45, 11.16, 10.33, 11.38, 7.77, 7.07, 8.0, 9.73, 7.60, 7.60, 7.73, 6.99, 2.15, 2.09, 1.99, 1.98 respectively, demonstrating transformer model has less value of MARE with input variables as T, T_max_, T_min_. Mean bias error for ANN models are less in comparison to conventional models, showing less error in ANN models.

## Electronic supplementary material

Below is the link to the electronic supplementary material.

Supplementary Material 1

## Data Availability

“The data used in this study is provided in the supplementary information file”.

## Abbreviations

DGSR: Daily global solar radiation (kWh/m^2^/day)
ANN: Artificial neural network
MNN: Modular neural network
RBFNN: Radial basis function neural network
TM: Transformer model
MARE: Mean absolute relative error
LSTMNN: Long short-term memory neural network
RNN: Recurrent neural networks
RF: Random forest
RMSPE: Root mean squared percentage error
MSRE: Mean squared relative error
RMSRE: Root mean squared relative error
SVM: Support vector machine
MSE: Mean square error
MBE: Mean bias error
RMSE: Root mean squared error
D: Day of the year
R: Correlation regression
E: Evaporation
RH: Relative humidity
SH: Sunshine hours
T_min_: Lowest temperature
T_max_: Highest temperature
R^2^: Coefficient of determination
WS: Wind speed
T: Average temperature
