# Supplementary material for: Comparative analysis of daily global solar radiation prediction using deep learning models inputted with stochastic variables
Source: Sci Rep. 2025 Mar 28;15:10786. doi: 10.1038/s41598-025-95281-7 (PMC11953364; doi:10.1038/s41598-025-95281-7)
Supplement: Supplementary file 1 — Supplementary Material 1 [file 41598_2025_95281_MOESM1_ESM.doc]

**Comparative analysis of daily global solar radiation prediction using deep learning models inputted with stochastic variables**

| 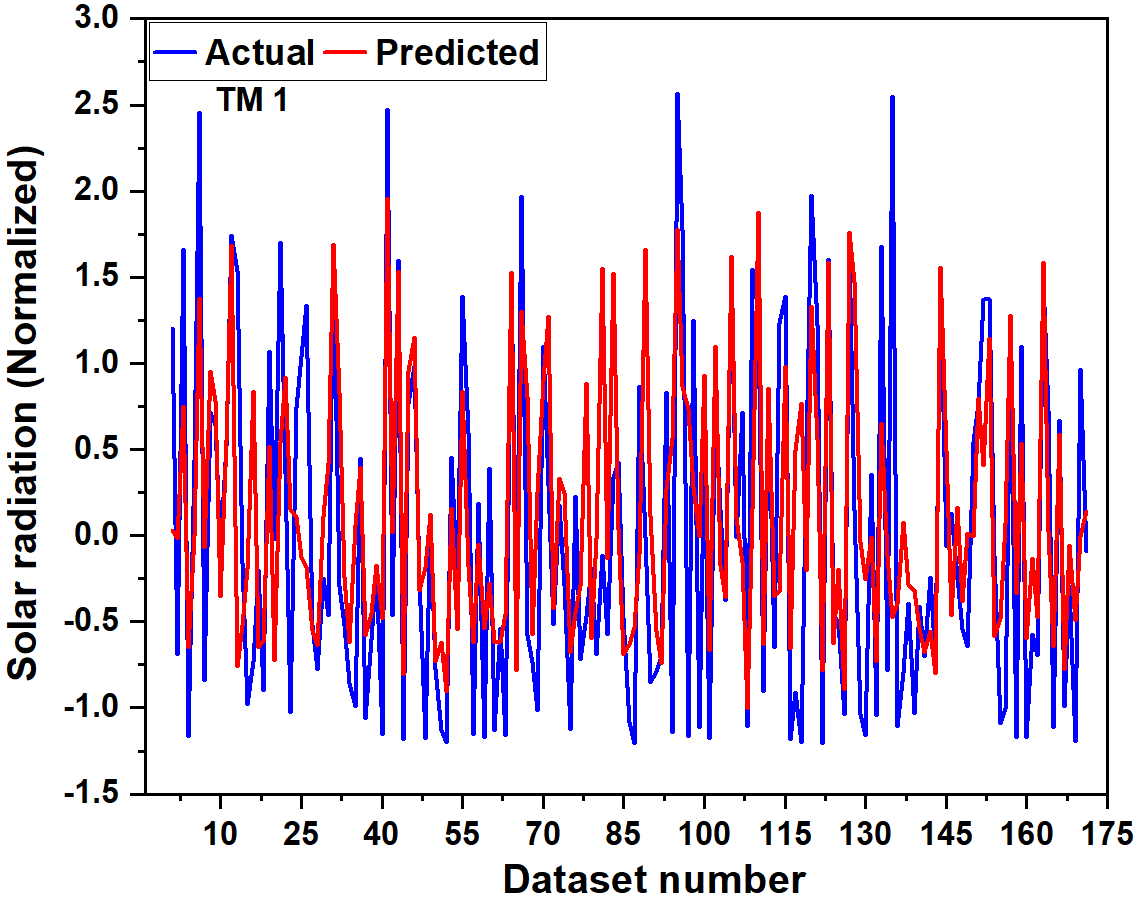 | 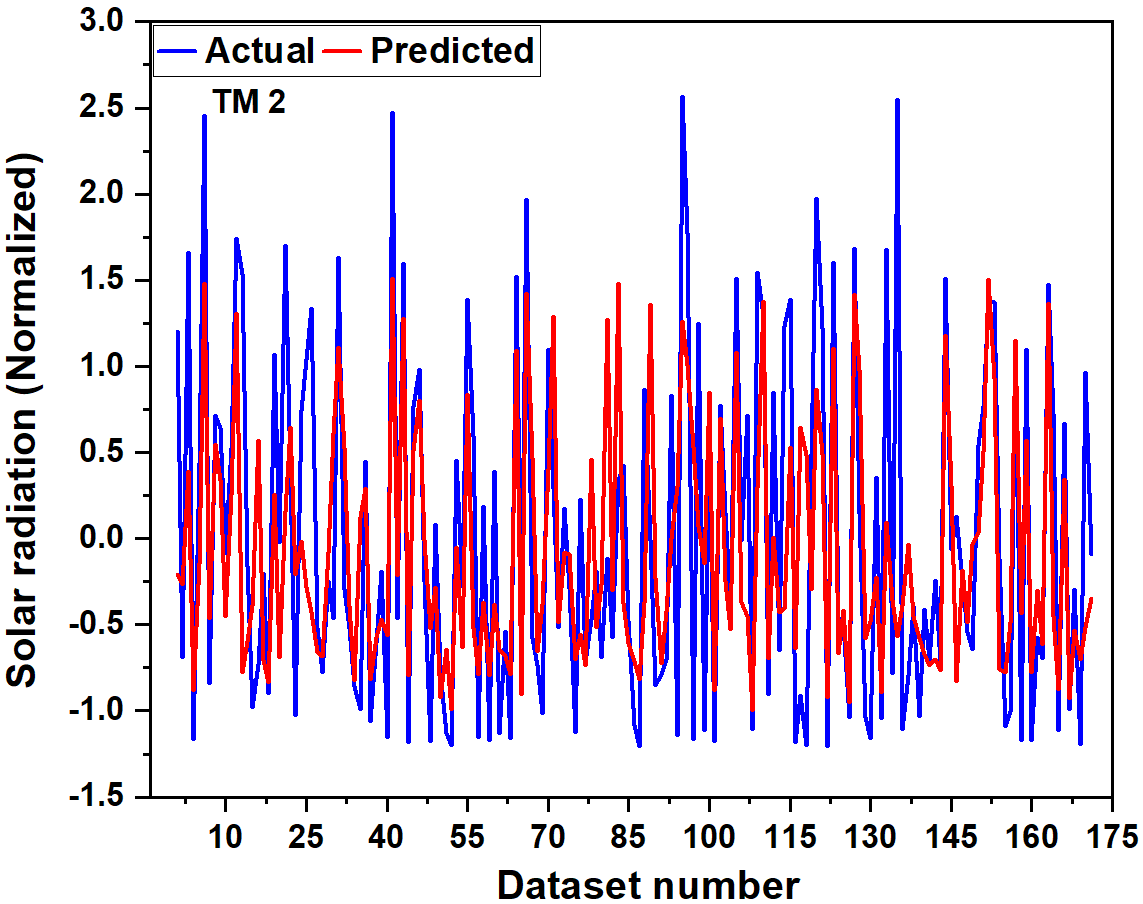  **(b)**  **(a)**  **(c)**  **(d)** |
| --- | --- |
| 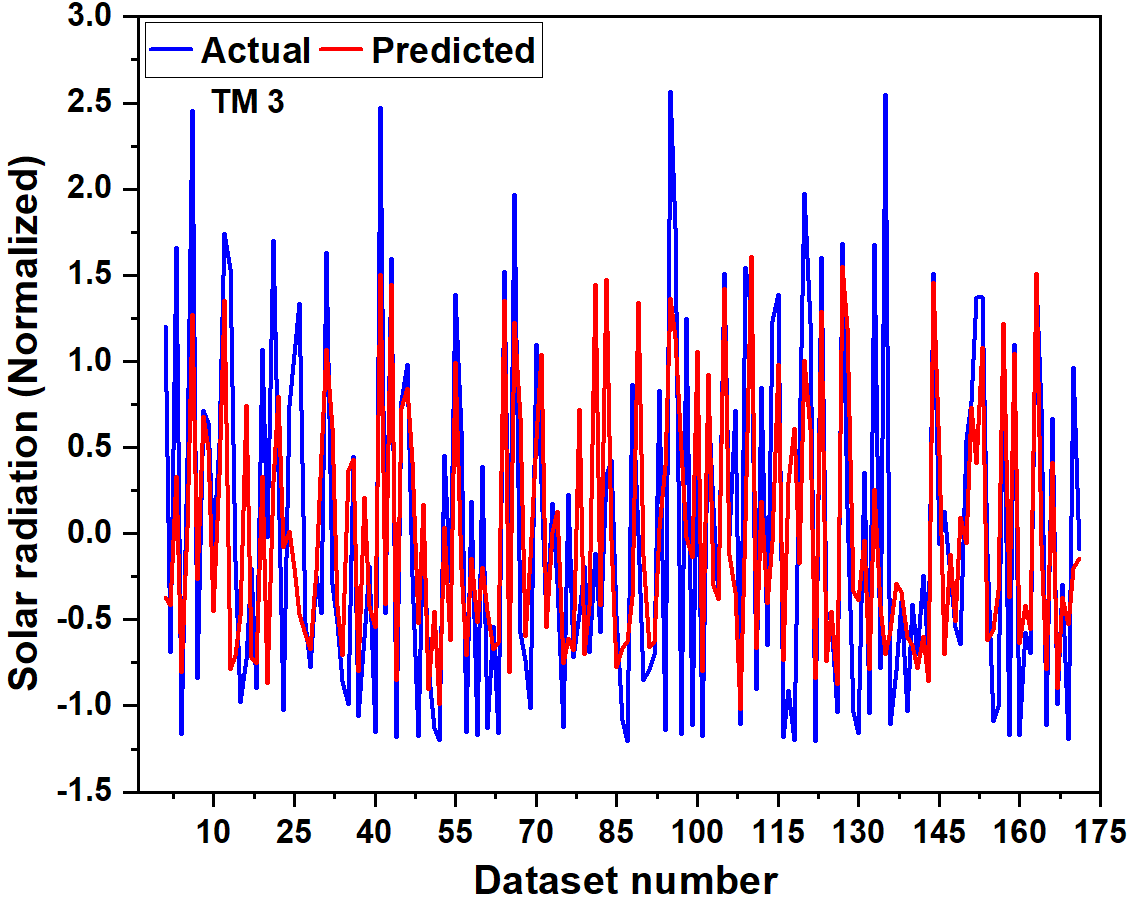 | 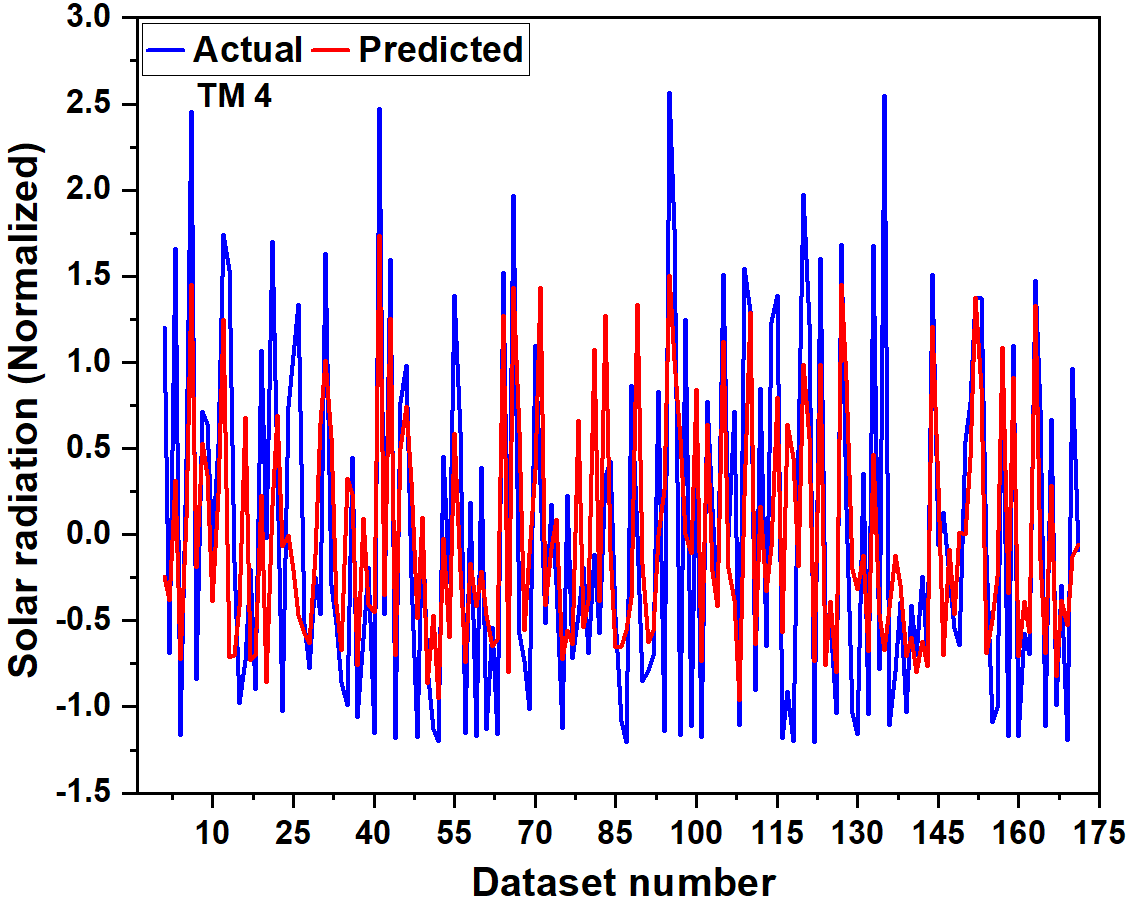 |

**Fig. S1.** Testing plots (a) TM-1, (b) TM-2, (c) TM-3, (d) TM-4.

| **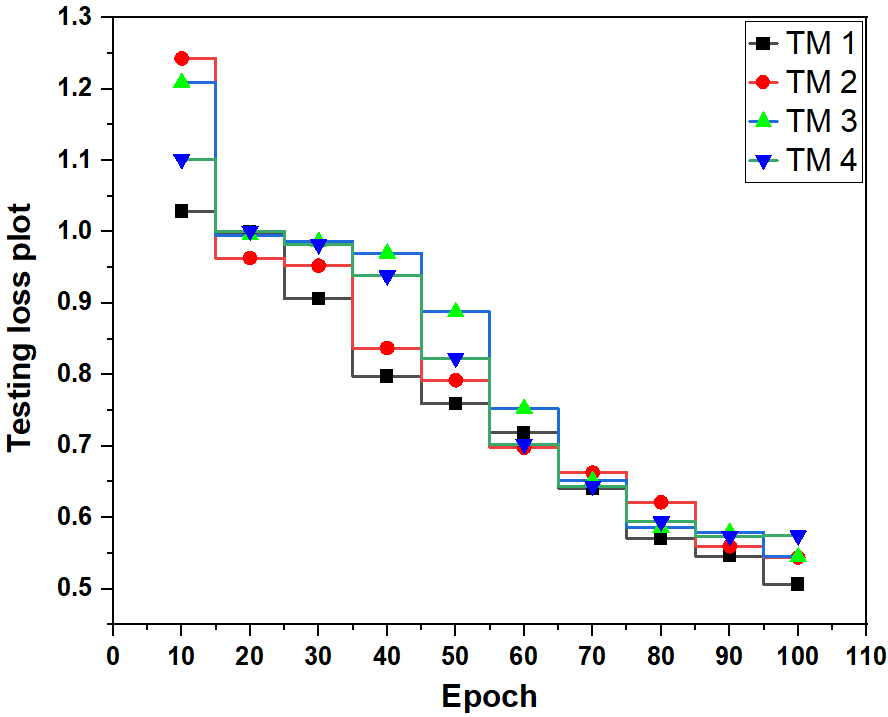** |
| --- |

**Fig. S2.** Testing loss plot for TM.

**Table S1:** Used datasets with input variables and target output.

| Input variables | | | | | Output |
| --- | --- | --- | --- | --- | --- |
| Wind speed | Relative humidity | Average temperature | Maximum temperature | Minimum temperature | Solar radiation |
| 1.7600 | 47.0391 | 24.8449 | 28.9400 | 20.4300 | 0.199000 |
| 1.3523 | 63.0051 | 22.6552 | 27.3500 | 18.4800 | 0.103800 |
| 1.3665 | 92.0997 | 20.3676 | 21.6700 | 17.5100 | 0.009100 |
| 2.2155 | 49.8675 | 21.9985 | 25.7600 | 19.9600 | 0.009600 |
| 1.6248 | 65.4145 | 22.3139 | 24.1900 | 20.2200 | 0.068300 |
| 1.7064 | 53.9372 | 23.6134 | 27.2500 | 18.2000 | 0.200200 |
| 2.0014 | 58.1430 | 21.2315 | 25.4900 | 18.5800 | 0.124700 |
| 3.5200 | 98.4162 | 14.7517 | 15.9300 | 12.3000 | 0.001800 |
| 1.7163 | 78.9356 | 20.2038 | 23.0800 | 17.1000 | 0.117800 |
| 1.9378 | 71.0903 | 18.7268 | 22.6100 | 14.2500 | 0.259400 |
| 1.8147 | 78.4496 | 17.1168 | 22.4700 | 14.5900 | 0.118400 |
| 1.7251 | 54.7772 | 20.3843 | 25.1900 | 16.3800 | 0.064500 |
| 2.0728 | 50.6882 | 21.3731 | 24.5800 | 16.3800 | 0.257500 |
| 2.2743 | 53.7209 | 20.0958 | 24.7500 | 14.0700 | 0.279500 |
| 1.9111 | 58.2266 | 18.5136 | 24.9500 | 15.5000 | 0.075500 |
| 2.0565 | 54.9582 | 20.9962 | 23.6200 | 18.5900 | 0.003900 |
| 2.9159 | 98.4039 | 18.0295 | 18.8000 | 16.4800 | 0.036300 |
| 1.9372 | 97.7643 | 18.5973 | 19.6300 | 17.8000 | 0.186300 |
| 2.4150 | 87.2448 | 20.2309 | 24.5700 | 17.0500 | 0.017300 |
| 1.9848 | 99.6421 | 17.1855 | 19.2100 | 16.0700 | 0.030700 |
| 1.5335 | 92.2322 | 17.4333 | 19.9200 | 16.0100 | 0.219500 |
| 1.8837 | 68.4559 | 19.2625 | 21.9400 | 15.4300 | 0.224200 |
| 1.4908 | 74.3326 | 18.2212 | 23.7000 | 14.4000 | 0.022000 |
| 2.3774 | 82.5861 | 17.4368 | 20.6600 | 15.7100 | 0.008000 |
| 1.3779 | 58.4353 | 20.6670 | 23.4100 | 18.0100 | 0.219900 |
| 2.5101 | 46.7337 | 23.8365 | 27.9400 | 20.0300 | 0.325800 |
| 1.9333 | 48.0759 | 23.0345 | 26.2000 | 18.6100 | 0.082000 |
| 2.0797 | 61.6331 | 19.0393 | 25.0900 | 16.7200 | 0.072000 |
| 2.0945 | 46.4686 | 23.4712 | 26.7900 | 17.7100 | 0.313500 |
| 2.3172 | 62.3999 | 21.1906 | 27.8400 | 17.4500 | 0.143000 |
| 1.7071 | 64.7312 | 21.0805 | 22.3100 | 19.2800 | 0.003500 |
| 2.2817 | 65.0245 | 22.7710 | 24.3400 | 21.1700 | 0.004800 |
| 3.1564 | 56.4719 | 23.5369 | 26.1800 | 21.5600 | 0.044500 |
| 2.1517 | 72.6943 | 23.6569 | 28.3000 | 18.2500 | 0.262900 |
| 1.0248 | 86.4091 | 21.7667 | 28.0400 | 18.8700 | 0.183000 |
| 1.4334 | 92.3634 | 18.6635 | 20.1600 | 17.0200 | 0.002100 |
| 2.0398 | 69.5595 | 19.1277 | 24.6000 | 16.0000 | 0.033100 |
| 1.9495 | 56.7353 | 18.8570 | 22.0700 | 16.2700 | 0.209600 |
| 2.5582 | 62.4570 | 19.1487 | 24.1600 | 12.8300 | 0.344700 |
| 2.1730 | 65.6093 | 18.6843 | 24.6300 | 15.5300 | 0.150800 |
| 2.2763 | 58.6920 | 18.4858 | 20.2700 | 15.7300 | 0.000025 |
| 2.2424 | 50.0229 | 23.1097 | 27.9300 | 19.7100 | 0.102500 |
| 3.0454 | 40.5503 | 24.9088 | 27.5400 | 22.0300 | 0.225100 |
| 4.1847 | 95.8894 | 18.8339 | 21.7200 | 17.9000 | 0.069500 |
| 2.1710 | 72.7817 | 20.8121 | 22.8600 | 18.7200 | 0.028700 |
| 1.7664 | 78.5396 | 19.4798 | 23.0500 | 17.7000 | 0.000015 |
| 2.1868 | 52.6273 | 20.6714 | 25.1500 | 16.9100 | 0.145300 |
| 2.0947 | 55.4862 | 22.7314 | 26.1900 | 17.3300 | 0.330100 |
| 1.8077 | 51.7024 | 22.1966 | 27.9300 | 16.9500 | 0.259100 |
| 1.7523 | 60.8414 | 21.2892 | 26.6300 | 18.9600 | 0.062700 |
| 2.0956 | 74.9312 | 21.2598 | 24.3700 | 18.9900 | 0.002200 |
| 2.1569 | 65.4706 | 22.3574 | 24.6000 | 19.5700 | 0.059200 |
| 2.4042 | 70.4083 | 24.9810 | 29.7400 | 19.2300 | 0.206300 |
| 1.9751 | 65.3032 | 25.1286 | 32.0100 | 21.5500 | 0.165200 |
| 2.0329 | 63.8303 | 25.5223 | 26.6600 | 23.1000 | 0.025500 |
| 1.8638 | 86.7226 | 24.6103 | 31.0300 | 20.4100 | 0.013100 |
| 2.5565 | 58.9969 | 25.6104 | 30.9700 | 21.0400 | 0.218700 |
| 2.1236 | 35.0050 | 18.0948 | 22.5000 | 11.3300 | 0.399100 |
| 1.5898 | 46.6889 | 15.6243 | 21.8900 | 10.5400 | 0.185600 |
| 1.9853 | 52.5346 | 14.2523 | 16.4700 | 12.5100 | 0.027300 |
| 2.5971 | 38.3759 | 17.5352 | 22.7500 | 12.0600 | 0.037400 |
| 2.3722 | 40.0434 | 20.4836 | 23.2700 | 17.4900 | 0.172200 |
| 2.9277 | 79.7836 | 16.5746 | 19.2900 | 12.8900 | 0.176300 |
| 1.7712 | 63.5587 | 16.4990 | 22.3300 | 13.7700 | 0.212600 |
| 1.5411 | 67.7441 | 13.8410 | 15.9700 | 11.6900 | 0.008000 |
| 2.5309 | 30.8864 | 16.1748 | 21.0400 | 12.8800 | 0.076600 |
| 2.9394 | 27.2997 | 16.4858 | 19.3000 | 12.9400 | 0.356600 |
| 1.8742 | 29.1690 | 15.2350 | 19.6100 | 9.6700 | 0.431000 |
| 1.4944 | 45.3502 | 12.6600 | 19.0000 | 9.7800 | 0.186900 |
| 1.5168 | 42.6722 | 12.7203 | 15.2000 | 10.5600 | 0.000986 |
| 2.6149 | 30.2746 | 17.1001 | 22.0000 | 13.7900 | 0.110100 |
| 1.9563 | 28.2093 | 20.2992 | 23.4900 | 16.5500 | 0.363700 |
| 3.3483 | 67.2854 | 13.8729 | 19.6100 | 7.4410 | 0.408100 |
| 1.8977 | 78.4335 | 12.6123 | 18.4300 | 11.0000 | 0.149400 |
| 2.2327 | 55.5780 | 13.2945 | 16.6600 | 10.7500 | 0.000176 |
| 3.1553 | 44.0064 | 17.6527 | 21.7500 | 14.1600 | 0.194500 |
| 1.9585 | 29.4383 | 22.1720 | 25.9600 | 15.5700 | 0.402700 |
| 1.9269 | 34.0939 | 21.8800 | 27.7300 | 13.9100 | 0.370500 |
| 1.8195 | 43.6980 | 19.9638 | 25.1800 | 16.9100 | 0.093700 |
| 1.9158 | 46.5635 | 22.1844 | 26.3500 | 20.0600 | 0.005400 |
| 2.1440 | 34.2553 | 26.6327 | 31.0800 | 23.4600 | 0.190800 |
| 4.0325 | 45.5530 | 23.4869 | 26.5200 | 16.3000 | 0.382200 |
| 2.4755 | 53.3267 | 14.2168 | 20.4600 | 10.6000 | 0.307500 |
| 2.3866 | 40.4342 | 12.6681 | 16.8100 | 11.5100 | 0.054400 |
| 2.9976 | 45.4622 | 16.2262 | 21.9400 | 14.2000 | 0.026400 |
| 2.8472 | 31.7709 | 22.0750 | 26.2000 | 18.2900 | 0.298700 |
| 2.8184 | 26.6697 | 24.5040 | 28.4100 | 17.3900 | 0.470400 |
| 2.0701 | 47.2798 | 20.5818 | 27.9900 | 15.9500 | 0.297200 |
| 1.7665 | 39.8012 | 27.2129 | 30.7000 | 21.3900 | 0.397100 |
| 2.0645 | 47.8883 | 24.7578 | 32.0600 | 20.0100 | 0.205700 |
| 2.2772 | 39.7857 | 23.9133 | 26.2900 | 21.3300 | 0.040800 |
| 2.2956 | 33.5611 | 24.8597 | 31.1200 | 20.6900 | 0.046800 |
| 2.7259 | 26.6320 | 28.2086 | 31.9200 | 22.9600 | 0.346900 |
| 3.0579 | 32.6463 | 25.0083 | 31.1400 | 18.0600 | 0.328100 |
| 2.0098 | 34.9264 | 21.8190 | 27.9800 | 17.2600 | 0.317600 |
| 1.8245 | 39.8762 | 21.5850 | 24.1400 | 18.9600 | 0.014600 |
| 2.3303 | 29.4555 | 24.9395 | 29.9600 | 20.3100 | 0.093500 |
| 3.0171 | 26.7775 | 27.9799 | 30.9000 | 24.4000 | 0.427100 |
| 2.3161 | 34.4546 | 26.4396 | 31.2800 | 20.4100 | 0.415600 |
| 2.2544 | 39.7578 | 23.5558 | 27.8400 | 18.9100 | 0.135000 |
| 2.6644 | 53.4327 | 19.5971 | 22.0100 | 14.1900 | 0.000121 |
| 4.4662 | 56.7926 | 18.3279 | 27.0600 | 13.6400 | 0.098400 |
| 2.1219 | 34.5303 | 24.4235 | 27.5300 | 20.6100 | 0.502400 |
| 2.5975 | 43.2188 | 20.8329 | 25.4900 | 17.2500 | 0.379200 |
| 4.2867 | 69.5832 | 16.6557 | 24.8600 | 12.9300 | 0.128500 |
| 2.5543 | 85.4285 | 14.6182 | 16.3500 | 13.3000 | 0.001500 |
| 2.7537 | 47.8283 | 20.7262 | 26.6000 | 15.0700 | 0.202900 |
| 1.8405 | 44.0500 | 21.8290 | 25.1500 | 17.8400 | 0.347000 |
| 1.6199 | 62.5869 | 19.6704 | 26.7900 | 16.4400 | 0.284600 |
| 1.6642 | 59.6765 | 19.7690 | 22.7700 | 16.9400 | 0.102600 |
| 1.6684 | 45.4279 | 23.2819 | 28.0100 | 20.8100 | 0.011100 |
| 2.3108 | 34.5246 | 27.3454 | 30.2800 | 24.7900 | 0.248300 |
| 2.5499 | 33.8351 | 28.5390 | 31.9300 | 23.8300 | 0.471300 |
| 2.2945 | 74.5534 | 20.7908 | 28.1200 | 15.6700 | 0.366800 |
| 1.7509 | 53.1218 | 19.3694 | 24.4100 | 17.2800 | 0.092900 |
| 3.0778 | 53.1500 | 19.4159 | 25.7100 | 16.8000 | 0.014500 |
| 2.7779 | 33.9652 | 25.5168 | 29.8800 | 20.9300 | 0.337400 |
| 1.9500 | 34.1257 | 26.2184 | 31.4200 | 18.7000 | 0.513600 |
| 2.1306 | 29.3406 | 24.1777 | 27.9700 | 17.9400 | 0.403000 |
| 2.1276 | 35.0480 | 21.5981 | 29.2800 | 16.9000 | 0.269300 |
| 1.5168 | 29.6709 | 22.0145 | 25.5500 | 19.6800 | 0.074900 |
| 2.1172 | 25.0009 | 25.6540 | 31.4800 | 21.2800 | 0.067400 |
| 2.2746 | 20.4797 | 28.9951 | 32.6000 | 24.5100 | 0.398400 |
| 2.2664 | 25.2254 | 27.8913 | 32.2500 | 21.3100 | 0.507400 |
| 2.2020 | 36.2889 | 23.9239 | 29.6600 | 19.1700 | 0.239000 |
| 1.9042 | 46.9837 | 23.7414 | 26.2600 | 20.6100 | 0.034300 |
| 2.6059 | 30.0113 | 28.1985 | 32.6200 | 25.1200 | 0.094100 |
| 3.0037 | 20.3086 | 32.2694 | 35.4900 | 29.0700 | 0.451900 |
| 2.4333 | 24.5478 | 31.2719 | 36.2500 | 24.8400 | 0.553400 |
| 2.2837 | 31.3774 | 26.6210 | 30.0500 | 21.2800 | 0.142500 |
| 1.5215 | 29.4537 | 27.6837 | 30.5900 | 24.1500 | 0.008600 |
| 3.9714 | 32.0630 | 27.7545 | 33.2800 | 21.0000 | 0.125800 |
| 2.7456 | 26.8906 | 29.5683 | 32.9000 | 23.2200 | 0.425400 |
| 2.1624 | 33.5444 | 27.7590 | 34.1000 | 21.2300 | 0.503800 |
| 2.0170 | 30.7449 | 25.8388 | 31.3700 | 22.4700 | 0.189900 |
| 1.9074 | 25.7751 | 26.4647 | 31.1500 | 23.6800 | 0.005300 |
| 3.2462 | 22.5124 | 29.3629 | 33.4800 | 23.5100 | 0.221700 |
| 3.0217 | 17.5052 | 32.7673 | 35.7800 | 27.4100 | 0.559000 |
| 2.0747 | 26.0500 | 29.0045 | 34.9100 | 24.6900 | 0.319100 |
| 1.4000 | 33.4598 | 26.7440 | 32.0000 | 23.0500 | 0.142200 |
| 2.3004 | 32.4649 | 27.9170 | 33.7800 | 23.9300 | 0.012700 |
| 3.1880 | 19.9238 | 30.3584 | 35.0900 | 25.7800 | 0.260200 |
| 2.1747 | 18.1041 | 31.0565 | 35.9200 | 24.2100 | 0.438900 |
| 3.8130 | 28.4529 | 26.6253 | 33.6600 | 22.1700 | 0.368000 |
| 2.5962 | 26.6119 | 26.4162 | 29.7700 | 22.4900 | 0.070700 |
| 2.5293 | 22.0015 | 28.2527 | 33.1200 | 24.7800 | 0.044900 |
| 3.4542 | 11.7192 | 32.8286 | 35.9400 | 28.5200 | 0.380000 |
| 3.5944 | 13.3206 | 33.1401 | 36.4400 | 25.6600 | 0.611600 |
| 2.1877 | 19.8503 | 30.8105 | 37.7600 | 25.8300 | 0.368400 |
| 2.7579 | 16.7107 | 36.1850 | 39.9400 | 29.5500 | 0.528200 |
| 2.2776 | 16.9587 | 33.1282 | 38.8000 | 28.8800 | 0.177900 |
| 1.9290 | 25.1671 | 27.8401 | 32.0500 | 25.3900 | 0.067900 |
| 3.1431 | 19.9707 | 30.9184 | 37.6700 | 25.9700 | 0.061700 |
| 3.3001 | 20.7298 | 33.5617 | 38.0200 | 27.4000 | 0.357100 |
| 2.6353 | 26.1667 | 32.0470 | 36.7600 | 25.3500 | 0.469300 |
| 3.0343 | 45.3713 | 24.7473 | 30.9400 | 21.4600 | 0.165800 |
| 2.2440 | 38.2494 | 25.1749 | 27.5500 | 22.6200 | 0.029200 |
| 2.7095 | 40.3830 | 25.5447 | 30.1000 | 21.9600 | 0.032100 |
| 2.5358 | 28.6493 | 31.4566 | 34.3400 | 27.3300 | 0.435100 |
| 2.5296 | 30.4866 | 30.5039 | 35.2700 | 23.2800 | 0.537700 |
| 1.8922 | 28.9351 | 28.2741 | 34.1900 | 24.8400 | 0.249900 |
| 3.0496 | 34.4017 | 26.4149 | 29.7100 | 23.7400 | 0.009800 |
| 1.7193 | 25.5219 | 30.7364 | 34.1700 | 27.2300 | 0.180700 |
| 3.3333 | 16.5246 | 35.3744 | 38.3200 | 31.9900 | 0.526400 |
| 2.6495 | 23.7209 | 32.3636 | 38.1300 | 25.5800 | 0.495000 |
| 1.3899 | 28.3215 | 30.0096 | 35.2600 | 26.6500 | 0.203700 |
| 2.0256 | 22.9112 | 31.1759 | 35.5400 | 28.1200 | 0.008600 |
| 3.1555 | 17.0998 | 35.0224 | 38.1800 | 31.0200 | 0.209200 |
| 3.6326 | 15.0312 | 36.7994 | 39.3400 | 31.1200 | 0.545000 |
| 2.4339 | 28.5686 | 33.6753 | 38.6400 | 28.9400 | 0.383300 |
| 1.7974 | 29.2343 | 31.3238 | 36.1200 | 29.7400 | 0.125800 |
| 1.2411 | 37.6479 | 29.1941 | 33.1100 | 25.5200 | 0.016000 |
| 2.8727 | 25.1900 | 33.7578 | 36.8000 | 30.6300 | 0.263300 |
| 3.4568 | 20.0550 | 35.4920 | 38.3600 | 29.0000 | 0.547700 |
| 2.3905 | 26.1386 | 33.1567 | 39.3700 | 29.1500 | 0.384400 |
| 1.8619 | 49.7074 | 30.8794 | 34.0800 | 29.3900 | 0.073900 |
| 3.4852 | 54.4805 | 28.7478 | 37.4000 | 22.8500 | 0.033400 |
| 3.3781 | 56.0904 | 28.7577 | 36.5500 | 20.2300 | 0.239800 |
| 2.0187 | 39.6094 | 31.9330 | 36.3600 | 26.4400 | 0.431900 |
| 2.8341 | 33.2678 | 35.2479 | 38.3700 | 29.5800 | 0.509600 |
| 2.1823 | 52.0504 | 31.3700 | 35.1600 | 28.5400 | 0.187000 |
| 1.6616 | 67.4574 | 28.4373 | 30.3500 | 26.0200 | 0.048800 |
| 2.0932 | 83.4780 | 26.4315 | 28.3100 | 21.9500 | 0.016100 |
| 3.0596 | 71.6937 | 28.1211 | 30.6700 | 25.1200 | 0.328500 |
| 2.7866 | 79.1198 | 25.1797 | 31.1600 | 19.0300 | 0.288500 |
| 1.4881 | 68.9527 | 27.5293 | 33.3700 | 24.7800 | 0.253700 |
| 2.1914 | 95.0107 | 23.4113 | 27.1700 | 20.7000 | 0.000291 |
| 2.3548 | 85.7966 | 25.4501 | 30.4200 | 21.6000 | 0.126200 |
| 2.2870 | 63.3912 | 28.8356 | 32.0000 | 25.7400 | 0.411700 |
| 1.7301 | 61.1185 | 29.3919 | 34.0100 | 21.8700 | 0.542100 |
| 1.2294 | 79.8358 | 27.1055 | 31.9600 | 23.6300 | 0.179400 |
| 1.4168 | 84.2702 | 26.0545 | 27.7200 | 24.6500 | 0.008700 |
| 2.0963 | 81.5769 | 26.0300 | 30.9000 | 23.6000 | 0.110700 |
| 1.9229 | 72.5089 | 28.6820 | 31.4400 | 24.4800 | 0.453800 |
| 1.6711 | 86.3280 | 25.1313 | 30.1600 | 22.3800 | 0.237300 |
| 1.7959 | 90.6574 | 24.0165 | 26.7600 | 22.4100 | 0.069800 |
| 1.5070 | 81.2785 | 23.2758 | 25.2400 | 20.8700 | 0.005100 |
| 1.5294 | 70.3729 | 27.7386 | 32.5600 | 24.9400 | 0.187900 |
| 1.8234 | 61.7218 | 29.2401 | 32.0400 | 26.2300 | 0.425000 |
| 2.1654 | 70.2319 | 28.2180 | 32.3700 | 22.8000 | 0.418900 |
| 1.1300 | 78.0495 | 27.2292 | 30.5900 | 25.5200 | 0.085300 |
| 2.9107 | 87.0206 | 24.3787 | 28.2100 | 21.5500 | 0.005300 |
| 1.9404 | 57.7034 | 30.4834 | 33.4100 | 26.6100 | 0.302400 |
| 1.9449 | 74.9527 | 27.2377 | 29.9200 | 22.1500 | 0.266500 |
| 1.6281 | 73.4270 | 26.9521 | 33.5900 | 22.9800 | 0.392200 |
| 1.3098 | 86.0404 | 26.0621 | 28.8100 | 24.5800 | 0.078500 |
| 1.0948 | 82.9038 | 26.9075 | 28.8800 | 25.6900 | 0.028800 |
| 2.1640 | 76.5458 | 29.4138 | 32.5400 | 26.4800 | 0.298900 |
| 1.5289 | 84.6049 | 27.1034 | 30.4100 | 22.7100 | 0.324100 |
| 2.0072 | 89.6102 | 24.9551 | 30.4400 | 21.8100 | 0.204600 |
| 1.4519 | 95.7891 | 24.6875 | 27.8000 | 21.8900 | 0.206900 |
| 1.4784 | 99.9813 | 23.5783 | 24.9500 | 22.3900 | 0.031900 |
| 2.2072 | 99.9601 | 23.8331 | 25.4700 | 22.3100 | 0.006400 |
| 1.4318 | 96.8683 | 25.6741 | 27.5200 | 24.6000 | 0.022000 |
| 3.3420 | 83.6689 | 25.4844 | 29.4800 | 20.7300 | 0.281400 |
| 1.4724 | 74.1113 | 26.7592 | 30.9100 | 20.2900 | 0.428500 |
| 1.5885 | 84.4927 | 25.8081 | 30.7200 | 23.0200 | 0.279300 |
| 0.7691 | 97.2873 | 25.2739 | 26.5200 | 24.0900 | 0.012600 |
| 1.6185 | 99.7019 | 23.8351 | 24.5700 | 22.8900 | 0.029900 |
| 1.6272 | 77.6026 | 27.5999 | 31.1600 | 24.8700 | 0.259300 |
| 1.6627 | 87.1087 | 26.0571 | 29.9400 | 22.8300 | 0.296900 |
| 1.8010 | 99.3648 | 23.2175 | 26.1000 | 22.0500 | 0.075400 |
| 1.7447 | 90.3138 | 23.4989 | 25.2500 | 21.6800 | 0.001900 |
| 1.8229 | 75.4178 | 26.5400 | 33.0000 | 22.6400 | 0.147900 |
| 2.0094 | 88.2107 | 24.5660 | 30.7200 | 21.6700 | 0.191400 |
| 2.0161 | 79.3139 | 25.6095 | 29.6100 | 22.0800 | 0.292300 |
| 1.5525 | 94.2536 | 23.7001 | 24.8600 | 21.9500 | 0.039400 |
| 2.4831 | 94.8014 | 23.7695 | 28.1600 | 21.7600 | 0.007000 |
| 1.6677 | 84.0092 | 26.8589 | 30.4700 | 24.7500 | 0.177800 |
| 1.8857 | 78.5026 | 28.1112 | 31.5000 | 25.7100 | 0.360100 |
| 1.4935 | 84.2839 | 27.5220 | 31.8100 | 24.1500 | 0.387300 |
| 1.9624 | 99.8012 | 23.3246 | 25.7600 | 21.5900 | 0.014100 |
| 1.9354 | 96.6958 | 24.9666 | 27.8600 | 22.4500 | 0.012400 |
| 1.4686 | 100.0000 | 23.2051 | 23.9500 | 22.5300 | 0.021700 |
| 2.2296 | 99.5832 | 23.3650 | 25.1600 | 22.1000 | 0.133400 |
| 1.3350 | 81.2524 | 22.6144 | 23.1700 | 22.0700 | 0.015300 |
| 1.3283 | 94.7100 | 22.4644 | 23.4600 | 21.8200 | 0.020300 |
| 1.9980 | 84.4927 | 22.9191 | 24.1600 | 21.9200 | 0.017500 |
| 2.2587 | 97.2873 | 26.7517 | 29.5500 | 23.8300 | 0.313300 |
| 1.2151 | 99.7019 | 24.2951 | 27.1600 | 22.1300 | 0.200500 |
| 1.7920 | 77.6026 | 23.0029 | 23.5900 | 22.1100 | 0.029500 |
| 1.7975 | 77.9689 | 27.8122 | 31.0500 | 22.9200 | 0.406800 |
| 1.3196 | 85.9657 | 26.2174 | 32.0000 | 23.7100 | 0.201800 |
| 2.0109 | 95.7737 | 23.6569 | 25.5100 | 21.5800 | 0.018600 |
| 1.3134 | 95.0366 | 23.7175 | 24.6100 | 22.0900 | 0.024200 |
| 2.2151 | 87.6704 | 25.2216 | 30.2500 | 22.5300 | 0.263400 |
| 2.2511 | 84.2607 | 25.1646 | 29.8100 | 21.4000 | 0.278700 |
| 1.5585 | 82.9470 | 24.7466 | 28.9800 | 21.9300 | 0.177100 |
| 1.9814 | 80.8730 | 24.4364 | 28.1300 | 22.9600 | 0.026100 |
| 1.8827 | 87.9129 | 25.2406 | 29.9100 | 20.1600 | 0.093700 |
| 1.7124 | 92.2198 | 24.0572 | 28.5600 | 22.3700 | 0.198100 |
| 1.6842 | 75.4627 | 27.0298 | 30.7200 | 22.6300 | 0.381500 |
| 1.6249 | 79.9255 | 25.4756 | 30.0700 | 23.0000 | 0.211400 |
| 1.4473 | 95.5956 | 24.6635 | 26.1400 | 23.4500 | 0.005200 |
| 1.9610 | 88.9374 | 25.5157 | 30.3900 | 23.4700 | 0.101800 |
| 2.1614 | 74.3906 | 27.9293 | 30.6300 | 25.5300 | 0.352400 |
| 1.3736 | 87.0653 | 25.6559 | 29.1400 | 22.4900 | 0.213900 |
| 1.9230 | 98.5134 | 22.5471 | 24.9000 | 21.1600 | 0.056800 |
| 1.5430 | 99.8428 | 23.1357 | 24.6200 | 21.7500 | 0.000277 |
| 1.6771 | 90.5421 | 25.0972 | 28.6100 | 22.7900 | 0.135500 |
| 1.3252 | 84.9735 | 25.4979 | 29.4400 | 22.4500 | 0.246100 |
| 1.7226 | 84.7598 | 23.8046 | 29.2200 | 19.8400 | 0.309300 |
| 3.4187 | 98.0540 | 20.1204 | 22.9800 | 18.1600 | 0.011600 |
| 1.5258 | 91.9878 | 21.0426 | 23.8800 | 19.8700 | 0.006200 |
| 2.2383 | 80.7936 | 24.8603 | 29.5400 | 21.7600 | 0.216200 |
| 2.3464 | 74.2857 | 25.4569 | 28.7800 | 20.1300 | 0.441400 |
| 1.6799 | 85.9476 | 23.6057 | 28.0600 | 20.4900 | 0.285900 |
| 1.3673 | 95.9693 | 22.2625 | 25.9700 | 20.7900 | 0.067100 |
| 1.9378 | 89.1723 | 23.2546 | 27.1300 | 21.1300 | 0.019300 |
| 2.3663 | 74.1222 | 25.0473 | 28.5300 | 22.2600 | 0.254500 |
| 2.4345 | 73.1414 | 25.2605 | 28.1000 | 20.2900 | 0.406500 |
| 1.5216 | 76.4500 | 22.8681 | 27.7600 | 18.7000 | 0.290500 |
| 2.1066 | 57.1827 | 26.8338 | 29.8600 | 20.9300 | 0.429600 |
| 1.4029 | 70.2477 | 24.2717 | 29.1900 | 20.5400 | 0.197100 |
| 0.8512 | 77.4493 | 22.8882 | 25.3100 | 21.2300 | 0.037200 |
| 2.1069 | 50.7249 | 22.9243 | 27.6600 | 19.5200 | 0.027400 |
| 2.1267 | 44.9786 | 26.0751 | 29.1800 | 21.7000 | 0.301400 |
| 2.1558 | 47.3004 | 25.3271 | 28.7300 | 19.5000 | 0.428000 |
| 1.7328 | 57.1974 | 22.8001 | 28.3000 | 19.3600 | 0.260600 |
| 1.3333 | 73.2293 | 21.6584 | 24.2400 | 19.8000 | 0.011400 |
| 2.2261 | 60.1111 | 23.1235 | 27.4400 | 20.0400 | 0.054400 |
| 2.1486 | 48.4466 | 25.4905 | 27.9500 | 22.8000 | 0.322100 |
| 1.9416 | 54.4929 | 24.0264 | 27.8500 | 17.2000 | 0.410800 |
| 1.6563 | 63.4320 | 21.2374 | 26.8800 | 18.6700 | 0.198000 |
| 1.8814 | 67.2101 | 20.4486 | 22.4700 | 18.3900 | 0.001800 |
| 2.0903 | 59.5789 | 22.7020 | 26.9300 | 19.0000 | 0.092400 |
| 1.6340 | 48.6308 | 25.1106 | 27.8600 | 22.2000 | 0.361100 |
| 1.6274 | 50.7655 | 22.9386 | 28.1900 | 16.4900 | 0.381600 |
| 1.3825 | 53.6060 | 20.7116 | 26.3700 | 18.3200 | 0.138900 |
| 1.3498 | 54.4641 | 21.1971 | 23.8800 | 18.7000 | 0.000000 |
| 3.0819 | 58.3677 | 19.0070 | 23.0900 | 15.9800 | 0.104400 |
| 1.9155 | 43.4221 | 23.4535 | 27.0800 | 19.7000 | 0.372100 |
| 1.5670 | 48.3457 | 21.2475 | 26.4100 | 16.5900 | 0.341600 |
| 1.6092 | 65.2161 | 18.3860 | 23.6600 | 16.5700 | 0.089200 |
| 1.8444 | 58.0759 | 19.4539 | 24.2000 | 14.2800 | 0.001200 |
| 1.8057 | 48.2172 | 22.5361 | 26.3200 | 18.6800 | 0.189200 |
| 1.5208 | 49.2194 | 21.8529 | 25.0700 | 17.1100 | 0.284600 |
| 1.4999 | 56.7341 | 20.0246 | 25.8200 | 14.8600 | 0.302800 |
| 1.4803 | 67.3494 | 18.1940 | 20.8300 | 15.8400 | 0.043800 |
| 1.6872 | 58.5563 | 19.2342 | 24.1200 | 14.5100 | 0.009600 |
| 2.2733 | 46.1260 | 21.2037 | 25.5000 | 12.6900 | 0.229400 |
| 2.0576 | 51.8131 | 19.1341 | 22.2700 | 13.1100 | 0.353200 |
| 1.1597 | 58.4169 | 18.1838 | 23.6400 | 13.9600 | 0.258800 |
| 1.6516 | 53.2461 | 22.1046 | 25.0300 | 15.1000 | 0.342800 |
| 1.5283 | 64.1915 | 19.4019 | 24.6300 | 16.1700 | 0.159200 |
| 1.7751 | 77.2444 | 17.4151 | 19.3700 | 15.4100 | 0.027300 |
| 1.6451 | 63.1548 | 19.0503 | 23.2000 | 16.3700 | 0.010900 |
| 2.0174 | 49.7057 | 21.2400 | 24.0200 | 18.4800 | 0.215800 |
| 2.0384 | 48.4978 | 20.5990 | 23.8800 | 15.4200 | 0.317100 |
| 1.6001 | 59.5743 | 18.1354 | 23.1500 | 14.1300 | 0.197600 |
| 1.7529 | 59.0527 | 16.7300 | 19.0500 | 14.7000 | 0.005800 |
| 2.0733 | 50.2205 | 18.1788 | 22.3700 | 15.1800 | 0.033900 |
| 2.4367 | 44.5043 | 20.0155 | 22.0900 | 17.5300 | 0.248800 |
| 1.6833 | 51.0402 | 19.0651 | 23.1400 | 14.2300 | 0.312700 |
| 1.9675 | 51.0738 | 16.8496 | 22.7900 | 14.7400 | 0.155100 |
| 1.9001 | 48.1003 | 16.5148 | 19.7500 | 14.5900 | 0.000194 |
| 1.1393 | 52.5362 | 17.7958 | 21.5000 | 14.2000 | 0.054900 |
| 1.7952 | 44.3939 | 19.7308 | 22.4000 | 16.2200 | 0.264900 |
| 1.5765 | 50.6599 | 18.2780 | 22.1000 | 13.6800 | 0.280500 |
| 1.4477 | 68.9659 | 15.3028 | 19.9600 | 11.9500 | 0.096000 |
| 1.9863 | 75.4440 | 14.7556 | 17.5400 | 11.9800 | 0.000000 |
| 2.1187 | 58.9807 | 17.6244 | 21.8500 | 13.5600 | 0.099200 |
| 1.5351 | 52.5249 | 19.5317 | 22.7400 | 14.6400 | 0.287400 |
| 1.3741 | 56.5231 | 17.7052 | 23.9300 | 12.4300 | 0.277800 |
| 1.5727 | 55.5329 | 15.4570 | 20.7500 | 12.9200 | 0.063700 |
| 1.5561 | 49.3958 | 16.3110 | 18.8000 | 14.0900 | 0.000166 |
| 2.4170 | 48.6683 | 18.4661 | 22.7300 | 14.6900 | 0.142200 |
| 2.0545 | 42.0692 | 18.6470 | 21.5200 | 13.7600 | 0.277600 |
| 1.2367 | 52.3532 | 16.0127 | 20.8900 | 13.3000 | 0.150900 |
| 1.5918 | 65.4725 | 13.6460 | 17.2100 | 10.9000 | 0.025800 |
| 1.6302 | 50.8566 | 15.2647 | 18.4700 | 13.2100 | 0.004500 |
| 1.8600 | 41.5206 | 16.9268 | 19.8000 | 13.7900 | 0.182700 |
| 1.8002 | 42.0009 | 17.0427 | 20.2500 | 12.7800 | 0.268400 |
| 1.5714 | 46.2117 | 16.3456 | 20.0100 | 9.8200 | 0.292300 |
| 1.8328 | 59.5806 | 12.8519 | 17.5900 | 10.4800 | 0.076100 |
| 1.6379 | 56.3197 | 11.9133 | 14.1500 | 9.7600 | 0.018300 |
| 2.0300 | 51.9744 | 13.5478 | 18.9600 | 10.4900 | 0.010900 |
| 1.8958 | 46.8589 | 17.3912 | 21.9800 | 13.1900 | 0.202800 |
| 1.8200 | 41.6766 | 17.5958 | 22.5500 | 11.5100 | 0.292200 |
| 1.3357 | 56.6070 | 14.2534 | 21.7300 | 9.4400 | 0.166800 |
| 2.5472 | 54.6726 | 13.5693 | 15.5500 | 9.9200 | 0.001300 |
| 1.4647 | 55.2391 | 15.6160 | 21.1200 | 12.7000 | 0.031300 |
| 1.4086 | 38.7114 | 19.0802 | 22.1100 | 15.4900 | 0.224100 |
| 1.6616 | 37.7367 | 18.8308 | 23.2700 | 13.8100 | 0.274000 |
| 2.0947 | 49.3121 | 14.9317 | 18.4400 | 13.5100 | 0.111400 |
| 2.4628 | 46.2505 | 14.5319 | 17.2300 | 9.4700 | 0.121400 |
| 1.9308 | 75.8768 | 10.0029 | 11.4500 | 8.3700 | 0.018600 |
| 1.3503 | 55.8774 | 13.9578 | 16.5000 | 10.3100 | 0.192200 |
| 2.0981 | 50.6804 | 13.1479 | 15.4500 | 10.5500 | 0.092600 |
| 2.6459 | 94.6033 | 9.2518 | 11.3500 | 8.3900 | 0.038300 |
| 1.9140 | 77.2379 | 11.6024 | 12.5900 | 10.4900 | 0.023400 |
| 2.6211 | 76.8169 | 12.1940 | 14.3600 | 10.4300 | 0.227800 |
| 1.6219 | 61.5531 | 15.7773 | 18.9800 | 11.8000 | 0.233100 |
| 1.7692 | 73.2773 | 13.2783 | 18.7500 | 8.5500 | 0.046400 |
| 2.2218 | 89.1381 | 12.0539 | 16.6200 | 10.2100 | 0.000140 |
| 2.3761 | 88.9771 | 11.9595 | 15.2200 | 9.8700 | 0.142500 |
| 2.7309 | 69.2098 | 13.5288 | 19.2100 | 9.9600 | 0.275000 |
| 2.1691 | 58.6364 | 15.0485 | 18.8500 | 10.6500 | 0.219000 |
| 1.2225 | 77.1201 | 12.1128 | 18.8600 | 7.6800 | 0.021800 |
| 1.4839 | 74.3651 | 10.5892 | 12.6100 | 6.6200 | 0.005400 |
| 1.9567 | 70.7084 | 11.9202 | 16.9700 | 8.6400 | 0.170200 |
| 2.2912 | 60.8854 | 13.1972 | 17.4100 | 9.5200 | 0.255700 |
| 1.8225 | 48.9359 | 15.4909 | 18.6800 | 9.5200 | 0.169900 |
| 1.5108 | 71.9649 | 12.3755 | 18.4900 | 8.4700 | 0.005500 |
| 2.6459 | 94.6033 | 9.2518 | 11.3500 | 8.3900 | 0.038300 |
| 1.6616 | 37.7367 | 18.8308 | 23.2700 | 13.8100 | 0.274000 |
| 1.5625 | 76.8776 | 10.8567 | 13.6800 | 6.5010 | 0.126800 |
| 1.8151 | 80.9545 | 8.3012 | 15.6000 | 4.3460 | 0.123600 |
| 1.5387 | 82.1115 | 7.5911 | 9.8700 | 5.7480 | 0.011400 |
| 1.7112 | 57.7837 | 9.9988 | 15.4900 | 6.8480 | 0.013200 |
| 1.7884 | 65.5315 | 11.7225 | 15.9800 | 6.5610 | 0.176000 |
| 1.7646 | 59.3358 | 10.9979 | 14.1800 | 6.4280 | 0.254900 |
| 1.2325 | 75.5676 | 6.7582 | 13.2000 | 2.6890 | 0.151600 |
| 1.0096 | 99.9230 | 4.0045 | 5.8410 | 2.5840 | 0.000825 |
| 2.2360 | 86.0842 | 6.4806 | 11.7100 | 3.7820 | 0.033800 |
| 1.9687 | 58.3829 | 11.0458 | 15.3000 | 5.7770 | 0.231800 |
| 2.0912 | 46.4409 | 12.3528 | 16.3700 | 6.4800 | 0.269600 |
| 1.9159 | 79.5743 | 8.8917 | 14.7900 | 6.0440 | 0.117400 |
| 2.1859 | 80.1917 | 8.5904 | 11.3600 | 5.4300 | 0.222000 |
| 1.8358 | 44.8428 | 14.2461 | 18.4600 | 11.0400 | 0.072500 |
| 1.8514 | 46.0355 | 16.2303 | 21.2900 | 13.4500 | 0.160600 |
| 1.8167 | 74.1130 | 14.2790 | 18.5600 | 10.0500 | 0.250900 |
| 1.1698 | 91.5830 | 12.0581 | 16.7000 | 10.8900 | 0.071800 |
| 1.8892 | 62.0124 | 10.8622 | 12.8000 | 9.1600 | 0.099200 |
| 2.3026 | 60.2980 | 13.0145 | 16.7000 | 11.1800 | 0.243900 |
| 1.4002 | 50.1361 | 15.5666 | 18.2100 | 9.8500 | 0.068200 |
| 2.5345 | 62.7564 | 12.1426 | 14.1000 | 9.8500 | 0.014700 |
| 3.4462 | 90.4929 | 9.1909 | 11.7900 | 7.4760 | 0.000032 |
| 2.9803 | 99.9594 | 6.3724 | 8.6000 | 5.2630 | 0.160900 |
| 2.2494 | 81.3638 | 10.3832 | 13.4600 | 7.6500 | 0.298100 |
| 2.1932 | 64.2973 | 11.5602 | 15.2000 | 4.4660 | 0.236800 |
| 1.3102 | 61.8859 | 11.3031 | 17.0600 | 6.7040 | 0.021800 |
| 1.6316 | 84.5939 | 9.2367 | 11.3600 | 7.9290 | 0.011200 |
| 1.6212 | 57.0472 | 11.1493 | 15.9600 | 8.7700 | 0.195900 |
| 2.4003 | 51.7138 | 12.7105 | 16.5200 | 9.0200 | 0.317700 |
| 1.9915 | 48.6759 | 13.7891 | 17.9600 | 7.3420 | 0.201500 |
| 1.7617 | 57.4464 | 11.2189 | 17.3900 | 6.7460 | 0.196800 |
| 1.5238 | 47.2082 | 16.2942 | 20.2700 | 9.0200 | 0.260900 |
| 1.7251 | 61.5164 | 14.2029 | 19.7500 | 10.7000 | 0.106000 |
| 1.8815 | 70.7647 | 12.6374 | 14.2300 | 10.7600 | 0.012100 |
| 1.9052 | 76.5363 | 13.0090 | 15.9200 | 10.5400 | 0.006600 |
| 2.2059 | 87.1327 | 12.5942 | 14.1400 | 10.2000 | 0.045500 |
| 3.2978 | 95.5501 | 11.4091 | 13.3800 | 10.1900 | 0.051700 |
| 2.6979 | 71.5330 | 9.5046 | 11.4400 | 8.1900 | 0.044500 |
| 1.5109 | 88.1580 | 7.9576 | 10.1100 | 5.3140 | 0.002800 |
| 2.6109 | 69.9929 | 11.6584 | 16.7200 | 7.8370 | 0.059600 |
| 2.1803 | 43.9916 | 16.7029 | 20.0700 | 10.8600 | 0.310600 |
| 1.4537 | 42.1565 | 16.1405 | 20.7200 | 8.8500 | 0.359800 |
| 1.6472 | 64.1343 | 12.6444 | 18.9300 | 8.9400 | 0.145200 |
| 1.5952 | 65.7983 | 14.7889 | 18.8300 | 11.2400 | 0.082500 |
| 2.2814 | 43.8913 | 17.0682 | 20.2600 | 13.2400 | 0.329000 |
| 2.0033 | 46.9470 | 16.1327 | 20.9600 | 10.6200 | 0.337600 |
| 1.6560 | 51.6248 | 13.7719 | 20.6400 | 10.2300 | 0.101500 |
| 2.1811 | 89.2312 | 10.8298 | 12.7200 | 9.8000 | 0.019900 |
| 1.8706 | 99.9916 | 9.6348 | 10.3800 | 9.1100 | 0.057800 |
| 1.5138 | 98.2318 | 10.2689 | 14.3700 | 8.7900 | 0.143400 |
| 1.9634 | 99.9832 | 9.4140 | 11.3500 | 8.1000 | 0.047300 |
| 2.2005 | 95.7852 | 10.9151 | 15.5700 | 9.7100 | 0.004400 |
| 2.0989 | 78.4776 | 13.6906 | 16.0700 | 11.2700 | 0.115500 |
| 2.2606 | 65.3319 | 15.6298 | 19.7700 | 9.0800 | 0.357300 |
| 1.4725 | 65.4238 | 14.1672 | 18.3300 | 10.4400 | 0.153800 |
| 3.0659 | 52.9898 | 13.8017 | 15.3000 | 12.2500 | 0.018000 |
| 3.2337 | 68.8697 | 13.6631 | 15.7100 | 10.0700 | 0.007300 |
| 2.4689 | 96.4232 | 10.5513 | 12.0300 | 9.7800 | 0.041400 |
| 1.4928 | 64.4294 | 15.9557 | 19.7600 | 8.1000 | 0.384500 |
| 1.7318 | 77.0340 | 14.4106 | 21.1500 | 10.4900 | 0.244800 |
| 2.1236 | 35.0050 | 18.0948 | 22.5000 | 11.3300 | 0.399100 |
| 1.5898 | 46.6889 | 15.6243 | 21.8900 | 10.5400 | 0.185600 |
| 1.9853 | 52.5346 | 14.2523 | 16.4700 | 12.5100 | 0.027300 |
| 2.5971 | 38.3759 | 17.5352 | 22.7500 | 12.0600 | 0.037400 |
| 2.3722 | 40.0434 | 20.4836 | 23.2700 | 17.4900 | 0.172200 |
| 2.9277 | 79.7836 | 16.5746 | 19.2900 | 12.8900 | 0.176300 |
| 1.7712 | 63.5587 | 16.4990 | 22.3300 | 13.7700 | 0.212600 |
| 1.5411 | 67.7441 | 13.8410 | 15.9700 | 11.6900 | 0.008000 |
| 2.5309 | 30.8864 | 16.1748 | 21.0400 | 12.8800 | 0.076600 |
| 2.9394 | 27.2997 | 16.4858 | 19.3000 | 12.9400 | 0.356600 |
| 1.8742 | 29.1690 | 15.2350 | 19.6100 | 9.6700 | 0.431000 |
| 1.4944 | 45.3502 | 12.6600 | 19.0000 | 9.7800 | 0.186900 |
| 1.5168 | 42.6722 | 12.7203 | 15.2000 | 10.5600 | 0.000986 |
| 2.6149 | 30.2746 | 17.1001 | 22.0000 | 13.7900 | 0.110100 |
| 1.9563 | 28.2093 | 20.2992 | 23.4900 | 16.5500 | 0.363700 |
| 3.3483 | 67.2854 | 13.8729 | 19.6100 | 7.4410 | 0.408100 |
| 1.8977 | 78.4335 | 12.6123 | 18.4300 | 11.0000 | 0.149400 |
| 2.2327 | 55.5780 | 13.2945 | 16.6600 | 10.7500 | 0.000176 |
| 3.1553 | 44.0064 | 17.6527 | 21.7500 | 14.1600 | 0.194500 |
| 1.9585 | 29.4383 | 22.1720 | 25.9600 | 15.5700 | 0.402700 |
| 1.9269 | 34.0939 | 21.8800 | 27.7300 | 13.9100 | 0.370500 |
| 1.8195 | 43.6980 | 19.9638 | 25.1800 | 16.9100 | 0.093700 |
| 1.9158 | 46.5635 | 22.1844 | 26.3500 | 20.0600 | 0.005400 |
| 2.1440 | 34.2553 | 26.6327 | 31.0800 | 23.4600 | 0.190800 |
| 4.0325 | 45.5530 | 23.4869 | 26.5200 | 16.3000 | 0.382200 |
| 2.4755 | 53.3267 | 14.2168 | 20.4600 | 10.6000 | 0.307500 |
| 2.3866 | 40.4342 | 12.6681 | 16.8100 | 11.5100 | 0.054400 |
| 2.9976 | 45.4622 | 16.2262 | 21.9400 | 14.2000 | 0.026400 |
| 2.8472 | 31.7709 | 22.0750 | 26.2000 | 18.2900 | 0.298700 |
| 2.8184 | 26.6697 | 24.5040 | 28.4100 | 17.3900 | 0.470400 |
| 2.0701 | 47.2798 | 20.5818 | 27.9900 | 15.9500 | 0.297200 |
| 1.7665 | 39.8012 | 27.2129 | 30.7000 | 21.3900 | 0.397100 |
| 2.0645 | 47.8883 | 24.7578 | 32.0600 | 20.0100 | 0.205700 |
| 2.2772 | 39.7857 | 23.9133 | 26.2900 | 21.3300 | 0.040800 |
| 2.2956 | 33.5611 | 24.8597 | 31.1200 | 20.6900 | 0.046800 |
| 2.7259 | 26.6320 | 28.2086 | 31.9200 | 22.9600 | 0.346900 |
| 3.0579 | 32.6463 | 25.0083 | 31.1400 | 18.0600 | 0.328100 |
| 2.0098 | 34.9264 | 21.8190 | 27.9800 | 17.2600 | 0.317600 |
| 1.8245 | 39.8762 | 21.5850 | 24.1400 | 18.9600 | 0.014600 |
| 2.3303 | 29.4555 | 24.9395 | 29.9600 | 20.3100 | 0.093500 |
| 3.0171 | 26.7775 | 27.9799 | 30.9000 | 24.4000 | 0.427100 |
| 2.3161 | 34.4546 | 26.4396 | 31.2800 | 20.4100 | 0.415600 |
| 2.2544 | 39.7578 | 23.5558 | 27.8400 | 18.9100 | 0.135000 |
| 2.6644 | 53.4327 | 19.5971 | 22.0100 | 14.1900 | 0.120500 |
| 4.4662 | 56.7926 | 18.3279 | 27.0600 | 13.6400 | 0.098400 |
| 2.1219 | 34.5303 | 24.4235 | 27.5300 | 20.6100 | 0.502400 |
| 2.5975 | 43.2188 | 20.8329 | 25.4900 | 17.2500 | 0.379200 |
| 4.2867 | 69.5832 | 16.6557 | 24.8600 | 12.9300 | 0.128500 |
| 2.5543 | 85.4285 | 14.6182 | 16.3500 | 13.3000 | 0.001500 |
| 2.7537 | 47.8283 | 20.7262 | 26.6000 | 15.0700 | 0.202900 |
| 1.8405 | 44.0500 | 21.8290 | 25.1500 | 17.8400 | 0.347000 |
| 1.6199 | 62.5869 | 19.6704 | 26.7900 | 16.4400 | 0.284600 |
| 1.6642 | 59.6765 | 19.7690 | 22.7700 | 16.9400 | 0.102600 |
| 1.6684 | 45.4279 | 23.2819 | 28.0100 | 20.8100 | 0.011100 |
| 2.3108 | 34.5246 | 27.3454 | 30.2800 | 24.7900 | 0.248300 |
| 2.5499 | 33.8351 | 28.5390 | 31.9300 | 23.8300 | 0.471300 |
| 2.2945 | 74.5534 | 20.7908 | 28.1200 | 15.6700 | 0.366800 |
| 1.7509 | 53.1218 | 19.3694 | 24.4100 | 17.2800 | 0.092900 |
| 3.0778 | 53.1500 | 19.4159 | 25.7100 | 16.8000 | 0.014500 |
| 2.7779 | 33.9652 | 25.5168 | 29.8800 | 20.9300 | 0.337400 |
| 1.9500 | 34.1257 | 26.2184 | 31.4200 | 18.7000 | 0.513600 |
| 2.1306 | 29.3406 | 24.1777 | 27.9700 | 17.9400 | 0.403000 |
| 2.3295 | 24.1787 | 29.6874 | 32.9300 | 24.2800 | 0.519900 |
| 1.9653 | 20.8766 | 25.5512 | 31.2500 | 21.6100 | 0.236700 |
| 1.7177 | 30.9305 | 23.4359 | 26.7300 | 21.1200 | 0.066400 |
| 1.7680 | 20.3707 | 27.8105 | 32.0700 | 23.9900 | 0.055200 |
| 1.9118 | 15.3650 | 31.4407 | 34.5300 | 28.0500 | 0.360500 |
| 2.6786 | 20.1122 | 30.7680 | 33.8900 | 26.4200 | 0.402500 |
| 2.1344 | 28.1281 | 27.3526 | 34.1800 | 23.6500 | 0.295500 |
| 1.7950 | 32.5198 | 24.6520 | 26.6500 | 22.8700 | 0.025600 |
| 2.2007 | 22.7752 | 29.8781 | 34.6600 | 24.5500 | 0.098100 |
| 3.3202 | 19.0243 | 32.4232 | 35.1900 | 28.4400 | 0.433700 |
| 2.3891 | 22.0240 | 30.7235 | 35.9200 | 21.2700 | 0.556000 |
| 1.5849 | 24.2451 | 28.1621 | 35.0400 | 24.9500 | 0.266700 |
| 3.7123 | 38.9875 | 25.4079 | 31.4100 | 22.3500 | 0.008900 |
| 4.0236 | 49.3644 | 22.2818 | 24.9900 | 18.5700 | 0.071900 |
| 2.8470 | 82.0993 | 18.6522 | 20.9700 | 16.5800 | 0.106800 |
| 1.7374 | 49.1009 | 24.6763 | 30.6100 | 17.3100 | 0.524200 |
| 1.9831 | 53.3653 | 22.9107 | 25.1900 | 19.5800 | 0.112900 |
| 1.8034 | 36.7156 | 24.9730 | 30.5000 | 22.0600 | 0.006200 |
| 2.9696 | 23.4161 | 30.4837 | 34.9900 | 23.4800 | 0.242500 |
| 3.0685 | 24.5197 | 31.7470 | 34.8100 | 27.4300 | 0.497700 |
| 2.4911 | 28.8897 | 29.0856 | 36.1400 | 23.1800 | 0.479300 |
| 2.0900 | 26.0904 | 28.4728 | 32.5900 | 25.6000 | 0.146500 |
| 1.9528 | 19.6114 | 31.8024 | 36.9200 | 27.6900 | 0.020000 |
| 3.2409 | 17.2260 | 35.3565 | 38.5100 | 32.0900 | 0.258300 |
| 2.5114 | 17.7455 | 35.5258 | 39.0300 | 28.6800 | 0.477200 |
| 2.4123 | 20.6862 | 32.8035 | 39.3000 | 27.4200 | 0.404500 |
| 2.0778 | 18.5429 | 31.2930 | 34.6200 | 29.1000 | 0.089900 |
| 2.1593 | 16.0234 | 34.5852 | 39.4500 | 30.2400 | 0.049800 |
| 2.4810 | 13.1898 | 37.8407 | 40.4600 | 34.7500 | 0.341100 |
| 2.8195 | 23.0690 | 36.0308 | 39.2900 | 29.4400 | 0.539300 |
| 2.3761 | 34.1998 | 31.9710 | 37.8400 | 28.7400 | 0.321500 |
| 2.4332 | 50.9774 | 29.8072 | 32.9300 | 25.3000 | 0.420700 |
| 1.7715 | 61.3948 | 27.4527 | 31.9100 | 24.4900 | 0.169100 |
| 1.6058 | 66.7405 | 26.6870 | 28.5900 | 25.0400 | 0.024700 |
| 1.9167 | 41.3285 | 29.6801 | 33.4100 | 25.8100 | 0.070900 |
| 2.5796 | 34.1540 | 33.7081 | 36.8700 | 29.7000 | 0.331700 |
| 2.6262 | 34.1438 | 34.0068 | 38.2300 | 27.2300 | 0.487800 |
| 2.1058 | 36.8639 | 31.0593 | 36.5100 | 27.8600 | 0.286800 |
| 1.5724 | 47.6270 | 26.2970 | 28.3100 | 22.8300 | 0.032200 |
| 2.6268 | 56.6856 | 29.2194 | 36.3800 | 23.3500 | 0.058500 |
| 1.5129 | 63.1663 | 29.5261 | 33.1000 | 25.2600 | 0.308700 |
| 1.5424 | 71.7622 | 29.3393 | 33.5700 | 25.4800 | 0.323200 |
| 1.7115 | 76.2576 | 28.6934 | 32.7300 | 26.4000 | 0.158400 |
| 1.7648 | 70.4995 | 29.8215 | 33.0100 | 26.9200 | 0.002400 |
| 3.1350 | 98.2351 | 22.5366 | 26.9300 | 20.7000 | 0.003700 |
| 2.3302 | 86.8113 | 25.1496 | 28.2500 | 22.7400 | 0.167700 |
| 2.6402 | 94.8729 | 22.4066 | 26.5500 | 20.2600 | 0.050600 |
| 2.5469 | 89.1323 | 23.4648 | 28.6100 | 20.6700 | 0.137800 |
| 3.3152 | 80.3036 | 23.3298 | 29.2700 | 18.8300 | 0.007000 |
| 2.8423 | 83.7084 | 20.2148 | 22.1800 | 17.7100 | 0.017900 |
| 1.6323 | 67.4392 | 26.4018 | 29.9600 | 23.6900 | 0.358700 |
| 2.0389 | 73.5418 | 24.9009 | 31.0400 | 19.5900 | 0.381800 |
| 1.9192 | 82.9875 | 21.0147 | 27.9300 | 18.6200 | 0.123900 |
| 2.4391 | 59.4342 | 25.3361 | 30.1200 | 22.3800 | 0.028500 |
| 2.4031 | 51.7867 | 29.0233 | 31.4200 | 26.3500 | 0.304800 |
| 1.4023 | 51.9037 | 31.0309 | 33.7800 | 25.6700 | 0.542400 |
| 1.2906 | 63.1087 | 29.3667 | 34.9000 | 26.2800 | 0.356400 |
| 1.8234 | 74.4140 | 27.8716 | 29.3600 | 25.8900 | 0.035300 |
| 1.7028 | 75.3199 | 28.9849 | 33.3900 | 24.7300 | 0.037800 |
| 1.6362 | 75.6657 | 29.5106 | 32.7200 | 24.1500 | 0.177400 |
| 2.4565 | 79.9066 | 27.8742 | 31.9700 | 21.9100 | 0.342800 |
| 2.1173 | 64.7823 | 29.5653 | 32.4800 | 24.5400 | 0.477900 |
| 1.4351 | 72.1141 | 28.2181 | 33.5700 | 25.1300 | 0.197200 |
| 2.3197 | 72.5754 | 25.3274 | 28.4900 | 22.7900 | 0.030200 |
| 1.4849 | 87.9336 | 27.2261 | 31.2500 | 25.5100 | 0.063200 |
| 1.4007 | 81.1159 | 27.9202 | 30.5200 | 23.6800 | 0.254300 |
| 1.4581 | 93.0090 | 25.8693 | 28.1800 | 23.9000 | 0.153100 |
| 1.4248 | 97.4698 | 24.3040 | 25.1600 | 22.9000 | 0.021100 |
| 1.6412 | 99.6245 | 23.6232 | 25.0900 | 21.9400 | 0.001700 |
| 1.9904 | 94.3198 | 25.5090 | 28.2000 | 23.9700 | 0.072600 |
| 1.3351 | 88.3404 | 25.6181 | 29.4700 | 21.0100 | 0.232100 |
| 2.1952 | 90.7924 | 25.1561 | 30.1400 | 21.9200 | 0.301700 |
| 2.4181 | 86.2699 | 23.8864 | 26.8500 | 20.4900 | 0.098800 |
| 1.1695 | 96.9575 | 23.5354 | 26.4800 | 21.7500 | 0.012100 |
| 1.8061 | 81.0201 | 28.0002 | 32.9100 | 24.7600 | 0.162700 |
| 2.3230 | 72.7134 | 29.0388 | 31.4400 | 26.3600 | 0.451500 |
| 1.8441 | 81.4206 | 27.3461 | 31.9500 | 23.7900 | 0.406300 |
| 0.8518 | 94.9025 | 26.1918 | 29.6800 | 24.5300 | 0.123100 |
| 1.6939 | 94.1385 | 25.4543 | 29.5400 | 23.2200 | 0.007000 |
| 1.4609 | 90.0314 | 27.5234 | 30.7800 | 25.6500 | 0.155700 |
| 1.6696 | 83.7952 | 27.2252 | 30.0700 | 23.3900 | 0.317500 |
| 1.4529 | 96.0672 | 25.1234 | 30.1100 | 22.4200 | 0.180900 |
| 1.5577 | 99.8083 | 25.0280 | 27.9600 | 24.2700 | 0.064600 |
| 1.3295 | 96.4852 | 25.8048 | 30.3400 | 23.5500 | 0.017300 |
| 1.3059 | 90.0619 | 27.8428 | 30.3900 | 23.4300 | 0.255600 |
| 1.4434 | 99.5148 | 23.2634 | 27.4400 | 20.7500 | 0.143900 |
| 2.1145 | 86.5268 | 25.7953 | 30.6100 | 22.3700 | 0.307600 |
| 1.3409 | 99.0543 | 24.4233 | 26.6700 | 22.9000 | 0.045700 |
| 1.6201 | 97.7644 | 25.2626 | 28.8200 | 22.5300 | 0.053000 |
| 1.4082 | 89.3515 | 26.5141 | 29.7600 | 23.5700 | 0.211200 |
| 1.8751 | 92.2481 | 25.9820 | 29.1300 | 22.4200 | 0.237800 |
| 2.2874 | 90.0938 | 23.7873 | 31.1000 | 19.4600 | 0.305900 |
| 1.7411 | 85.0368 | 27.4413 | 31.1700 | 23.5200 | 0.362300 |
| 2.4853 | 99.5319 | 23.6694 | 26.1800 | 22.0900 | 0.050200 |
| 1.6473 | 92.1704 | 24.6720 | 28.3400 | 23.4200 | 0.044100 |
| 2.4952 | 90.5458 | 25.3529 | 29.9700 | 22.8600 | 0.074900 |
| 2.2937 | 81.5633 | 27.9839 | 31.5500 | 24.1500 | 0.299600 |
| 1.3501 | 100.0000 | 24.6302 | 25.9800 | 23.3300 | 0.104000 |
| 1.8210 | 99.9036 | 23.6984 | 26.1700 | 22.6500 | 0.063900 |
| 1.4236 | 97.8701 | 22.6030 | 23.3100 | 22.2800 | 0.006700 |
| 1.3385 | 87.6242 | 25.0525 | 27.5200 | 23.5400 | 0.052000 |
| 1.4862 | 97.5906 | 27.1114 | 30.3900 | 23.0600 | 0.289600 |
| 1.6570 | 96.4137 | 24.0093 | 27.0400 | 21.9600 | 0.148600 |
| 1.0098 | 99.2271 | 24.5571 | 30.1900 | 22.9300 | 0.121900 |
| 0.8483 | 94.4100 | 24.8257 | 26.5400 | 24.1000 | 0.010100 |
| 1.3357 | 95.3346 | 25.0833 | 29.8100 | 23.0500 | 0.112200 |
| 1.6666 | 91.4556 | 25.5245 | 27.8400 | 23.9500 | 0.190100 |
| 1.8730 | 92.2813 | 25.5337 | 28.7400 | 23.1700 | 0.299800 |
| 1.6710 | 99.9442 | 24.2907 | 27.4500 | 22.4300 | 0.111200 |
| 1.8497 | 94.0246 | 22.5082 | 23.2500 | 21.8500 | 0.001100 |
| 1.4988 | 97.8899 | 22.8222 | 23.5900 | 22.2700 | 0.059100 |
| 2.0813 | 98.0942 | 24.5927 | 28.7800 | 21.9900 | 0.256100 |
| 1.6055 | 98.5361 | 23.8940 | 27.3600 | 21.8000 | 0.167600 |
| 1.2254 | 90.5735 | 23.8457 | 27.4800 | 22.7200 | 0.085600 |
| 1.6925 | 82.9383 | 23.6866 | 25.2200 | 22.0800 | 0.019800 |
| 1.0078 | 85.9593 | 26.0258 | 29.4100 | 23.2600 | 0.141200 |
| 1.5769 | 93.0040 | 27.0556 | 30.1300 | 23.2700 | 0.375500 |
| 1.7114 | 96.8537 | 25.9064 | 31.7600 | 21.7700 | 0.345300 |
| 1.5854 | 91.4996 | 23.5589 | 26.3600 | 21.8900 | 0.064600 |
| 1.7107 | 82.1009 | 24.1596 | 27.1500 | 22.3200 | 0.023300 |
| 1.3402 | 88.8605 | 26.5987 | 30.6300 | 23.9300 | 0.146100 |
| 2.0097 | 97.0942 | 27.5075 | 30.4400 | 22.8900 | 0.361900 |
| 1.3059 | 91.4556 | 25.9685 | 30.0800 | 23.2300 | 0.204000 |
| 2.1671 | 74.8924 | 27.4591 | 30.3300 | 21.1300 | 0.464500 |
| 1.2705 | 90.0673 | 25.2464 | 30.0700 | 22.6000 | 0.194600 |
| 0.9325 | 99.5145 | 23.5403 | 25.6900 | 22.0600 | 0.030700 |
| 1.5815 | 86.3203 | 25.0605 | 30.2500 | 22.9600 | 0.045100 |
| 2.1645 | 66.8553 | 26.6247 | 31.0800 | 23.0100 | 0.297700 |
| 2.0564 | 73.5892 | 25.4204 | 30.2900 | 18.2800 | 0.372500 |
| 1.6356 | 81.2758 | 23.6516 | 29.0300 | 20.1300 | 0.261000 |
| 1.3846 | 93.9124 | 22.2192 | 24.2200 | 20.9100 | 0.010400 |
| 1.9583 | 94.9260 | 23.4434 | 28.6000 | 21.5400 | 0.054100 |
| 1.7570 | 88.5862 | 23.4337 | 28.0600 | 18.7500 | 0.217600 |
| 1.7943 | 74.6810 | 25.0312 | 28.7400 | 21.3000 | 0.342700 |
| 1.4860 | 78.1555 | 23.6802 | 28.6500 | 21.5200 | 0.210300 |
| 1.4344 | 96.6476 | 22.7436 | 24.3000 | 21.1300 | 0.000639 |
| 2.2764 | 83.9939 | 24.9862 | 28.8700 | 21.6000 | 0.130200 |
| 1.8659 | 72.1100 | 27.4502 | 29.9500 | 24.7700 | 0.335700 |
| 1.5298 | 76.8555 | 25.9987 | 30.6400 | 21.5100 | 0.385300 |
| 1.4611 | 91.2845 | 23.8857 | 30.1500 | 22.0400 | 0.148200 |
| 1.5519 | 93.0450 | 23.6037 | 26.1500 | 21.5200 | 0.000374 |
| 2.7538 | 75.8703 | 22.3458 | 30.5400 | 16.8300 | 0.109900 |
| 2.1584 | 60.1105 | 25.2218 | 29.2800 | 20.1300 | 0.382200 |
| 1.6965 | 57.6476 | 25.1190 | 30.2400 | 19.5100 | 0.415900 |
| 1.6911 | 74.7460 | 22.2541 | 27.9400 | 20.0200 | 0.113400 |
| 1.8831 | 89.9494 | 23.0981 | 27.7700 | 21.1600 | 0.007200 |
| 1.4828 | 73.7504 | 27.1577 | 30.9400 | 23.4600 | 0.238400 |
| 1.7714 | 64.0427 | 28.4528 | 31.3300 | 23.2000 | 0.436500 |
| 1.6296 | 84.5606 | 24.3268 | 29.5700 | 20.3300 | 0.224200 |
| 1.6097 | 89.8310 | 22.7318 | 23.8000 | 21.6600 | 0.020100 |
| 1.7108 | 88.7868 | 24.2415 | 29.0400 | 22.1100 | 0.019900 |
| 1.5652 | 81.5490 | 26.8898 | 30.0800 | 24.5400 | 0.207900 |
| 1.7297 | 84.5235 | 25.6327 | 29.7100 | 22.9300 | 0.210400 |
| 1.5364 | 82.8948 | 24.4967 | 27.6600 | 21.6100 | 0.258900 |
| 1.3203 | 82.4288 | 23.4915 | 29.3700 | 20.7000 | 0.154500 |
| 0.9127 | 94.4742 | 22.2609 | 23.7700 | 20.8400 | 0.025000 |
| 1.7806 | 90.0516 | 22.7382 | 26.8100 | 20.7600 | 0.023300 |
| 1.8301 | 86.0787 | 23.6457 | 26.8300 | 21.4000 | 0.158000 |
| 2.0580 | 84.5036 | 23.6547 | 27.4400 | 19.6400 | 0.334400 |
| 1.1465 | 93.1516 | 22.7934 | 27.7700 | 20.5600 | 0.171100 |
| 1.1669 | 100.0000 | 22.0164 | 22.7900 | 21.3200 | 0.009400 |
| 1.8969 | 96.6357 | 24.0194 | 27.3800 | 22.5700 | 0.040200 |
| 1.6151 | 96.1377 | 24.4177 | 26.0100 | 23.2600 | 0.126800 |
| 2.2178 | 88.1916 | 24.3756 | 27.2700 | 20.9800 | 0.243500 |
| 1.0719 | 97.3570 | 23.0389 | 26.8000 | 21.5900 | 0.099400 |
| 1.3556 | 85.6858 | 22.8163 | 24.0200 | 21.3200 | 0.000902 |
| 2.3363 | 79.3674 | 20.8170 | 23.1400 | 19.2400 | 0.035900 |
| 1.8310 | 62.6855 | 22.7730 | 25.0300 | 20.7100 | 0.155200 |
| 1.5267 | 75.9606 | 24.1466 | 27.7200 | 21.0700 | 0.241900 |
| 1.4783 | 80.2777 | 21.7646 | 26.4300 | 20.0000 | 0.120600 |
| 1.5341 | 65.7908 | 21.0663 | 23.1200 | 19.2300 | 0.000000 |
| 1.9616 | 59.7943 | 23.6432 | 26.9800 | 20.1200 | 0.136300 |
| 1.9959 | 65.4888 | 25.1230 | 27.7400 | 20.8700 | 0.371800 |
| 1.9443 | 86.3732 | 22.7438 | 28.0000 | 16.9200 | 0.346400 |
| 1.4730 | 69.7818 | 19.8066 | 24.6000 | 17.3900 | 0.084000 |
| 1.8716 | 51.0369 | 21.0932 | 25.1000 | 18.7600 | 0.001200 |
| 2.2357 | 54.3120 | 23.5892 | 27.0800 | 20.5700 | 0.187000 |
| 2.1576 | 64.8765 | 23.1711 | 25.6400 | 18.6700 | 0.360000 |
| 1.6585 | 75.3970 | 20.8363 | 25.3300 | 17.5200 | 0.267100 |
| 1.4822 | 77.1827 | 19.2087 | 21.9500 | 17.8400 | 0.040700 |
| 1.7742 | 64.8827 | 20.5898 | 24.4100 | 18.1200 | 0.008700 |
| 1.3960 | 69.4838 | 22.8580 | 26.1700 | 19.6100 | 0.163700 |
| 2.1554 | 82.3172 | 22.2882 | 25.5400 | 17.9900 | 0.299800 |
| 1.6325 | 74.3970 | 20.0217 | 25.8300 | 16.7800 | 0.214400 |
| 1.6121 | 54.2735 | 20.1099 | 23.6900 | 15.6900 | 0.254900 |
| 1.5854 | 71.4684 | 17.5487 | 22.3800 | 13.7800 | 0.136600 |
| 1.2065 | 66.8120 | 15.8379 | 17.9900 | 13.7500 | 0.024800 |
| 1.9381 | 53.5702 | 17.2775 | 22.0400 | 14.8700 | 0.013000 |
| 2.2796 | 46.1888 | 19.7391 | 22.8700 | 16.4200 | 0.215100 |
| 1.3237 | 56.8335 | 18.9302 | 22.8800 | 13.0000 | 0.234700 |
| 1.6618 | 66.9655 | 16.9197 | 21.8700 | 13.7000 | 0.166100 |
| 1.6075 | 63.1251 | 16.8002 | 18.0800 | 14.5400 | 0.003400 |
| 2.9916 | 89.4208 | 12.6421 | 19.1500 | 10.6000 | 0.011000 |
| 2.3391 | 52.0431 | 17.9512 | 22.0300 | 14.1400 | 0.240600 |
| 1.8229 | 53.7229 | 16.6272 | 20.0700 | 12.2500 | 0.289000 |
| 1.4124 | 61.6588 | 14.5729 | 22.3800 | 10.8100 | 0.154400 |
| 1.7614 | 58.4105 | 14.8593 | 17.0800 | 13.2000 | 0.000198 |
| 1.8799 | 49.9903 | 16.6333 | 20.9600 | 12.7800 | 0.065800 |
| 1.7067 | 46.3661 | 18.9542 | 21.9300 | 15.8100 | 0.281300 |
| 1.9674 | 45.4328 | 17.4043 | 21.5700 | 13.1400 | 0.300200 |
| 1.2546 | 56.9973 | 14.8067 | 20.3500 | 12.5900 | 0.108100 |
| 2.1210 | 43.4776 | 17.9101 | 22.3900 | 13.8000 | 0.105600 |
| 1.6795 | 42.7868 | 19.5879 | 23.0400 | 14.6100 | 0.293600 |
| 1.4971 | 53.9986 | 17.0384 | 21.4900 | 12.8100 | 0.273900 |
| 1.7001 | 65.8239 | 14.4191 | 20.7000 | 11.6500 | 0.063900 |
| 2.6146 | 60.6475 | 14.9039 | 18.3500 | 11.7700 | 0.000172 |
| 2.2487 | 51.3735 | 17.7709 | 21.8300 | 13.0600 | 0.140600 |
| 1.6604 | 46.5223 | 18.7934 | 22.3900 | 12.6300 | 0.293100 |
| 2.0709 | 56.7350 | 15.7852 | 20.6100 | 11.8200 | 0.227100 |
| 1.6029 | 65.4357 | 14.0035 | 16.1100 | 9.7600 | 0.027500 |
| 1.8117 | 63.3819 | 15.5548 | 19.0200 | 13.6200 | 0.004200 |
| 2.3491 | 54.0980 | 18.3049 | 21.3700 | 15.2700 | 0.177600 |
| 1.8952 | 49.4065 | 19.7518 | 23.1900 | 13.1900 | 0.267200 |
| 1.5996 | 62.8395 | 17.2844 | 22.3500 | 13.6700 | 0.188200 |
| 1.8956 | 46.2439 | 17.2076 | 20.7700 | 13.1400 | 0.254800 |
| 1.5785 | 53.4185 | 15.0192 | 20.1800 | 12.3300 | 0.103500 |
| 1.8911 | 58.9090 | 13.5125 | 15.4200 | 12.1700 | 0.012100 |
| 2.3534 | 57.4999 | 14.8817 | 18.5500 | 12.9500 | 0.007300 |
| 2.4315 | 41.4685 | 17.6062 | 20.7000 | 13.6200 | 0.171200 |
| 1.5657 | 42.6845 | 17.7267 | 22.0400 | 12.7300 | 0.256400 |
| 1.6173 | 59.6738 | 13.5865 | 19.7100 | 10.3500 | 0.098400 |
| 1.7404 | 59.7648 | 13.6386 | 15.9700 | 10.1100 | 0.003200 |
| 1.6560 | 60.2349 | 15.6027 | 20.3000 | 12.6600 | 0.033400 |
| 1.7641 | 50.7343 | 17.5467 | 19.9900 | 14.0600 | 0.200600 |
| 1.8659 | 50.6044 | 16.3364 | 19.7900 | 11.7000 | 0.236900 |
| 1.6200 | 66.6376 | 13.1826 | 19.3300 | 9.1800 | 0.111900 |
| 2.3951 | 63.2790 | 12.9712 | 15.5900 | 10.3500 | 0.000006 |
| 2.7205 | 54.5623 | 14.8954 | 19.6100 | 12.5800 | 0.055600 |
| 2.7215 | 53.3107 | 15.9435 | 19.3000 | 11.4900 | 0.231200 |
| 1.8126 | 59.4935 | 14.7530 | 19.1700 | 8.6400 | 0.245600 |
| 2.0461 | 73.2148 | 12.0435 | 17.4400 | 9.3900 | 0.076200 |
| 2.2390 | 77.7658 | 11.8723 | 14.2400 | 9.8000 | 0.176200 |
| 1.3776 | 60.1399 | 15.2354 | 18.4800 | 12.1600 | 0.078200 |
| 1.8689 | 56.3421 | 15.9430 | 18.5900 | 11.1800 | 0.225300 |
| 1.4526 | 74.1440 | 13.9749 | 18.8800 | 10.3700 | 0.189300 |
| 1.5666 | 94.4956 | 11.4655 | 14.2600 | 9.9700 | 0.035000 |
| 1.5367 | 85.6178 | 11.8510 | 13.6400 | 8.5200 | 0.000096 |
| 1.7192 | 55.5841 | 14.1499 | 17.2900 | 10.9400 | 0.113300 |
| 1.7386 | 48.9520 | 14.2926 | 17.2600 | 9.8900 | 0.219100 |
| 1.9181 | 51.6033 | 12.2113 | 16.7700 | 8.5800 | 0.151900 |
| 3.1470 | 99.9792 | 7.8847 | 8.4900 | 7.0380 | 0.005400 |
| 2.7962 | 85.8335 | 10.1221 | 12.9300 | 8.9000 | 0.003100 |
| 1.9471 | 72.3575 | 13.4808 | 17.3700 | 9.3200 | 0.147100 |
| 1.9654 | 81.8077 | 12.3932 | 15.5600 | 8.0200 | 0.236500 |
| 1.6389 | 92.6107 | 9.8116 | 15.6400 | 5.6390 | 0.163800 |
| 1.4579 | 95.8145 | 10.7723 | 14.2900 | 8.3500 | 0.178700 |
| 1.3815 | 98.7488 | 8.4999 | 15.6200 | 6.2520 | 0.063500 |
| 2.0860 | 98.0705 | 9.2185 | 12.2900 | 5.8290 | 0.053500 |
| 2.1803 | 57.3259 | 13.7642 | 17.7400 | 9.1900 | 0.166400 |
| 1.7454 | 63.9545 | 10.9466 | 14.6300 | 8.0100 | 0.125800 |
| 1.6660 | 88.1189 | 9.7689 | 14.4500 | 7.7530 | 0.045300 |
| 2.5995 | 54.1525 | 12.9580 | 15.7200 | 9.9600 | 0.211500 |
| 1.1401 | 64.8522 | 10.4763 | 14.4100 | 5.4620 | 0.189300 |
| 1.4961 | 62.3623 | 9.0219 | 10.1000 | 8.0200 | 0.004400 |
| 1.8806 | 66.7908 | 10.4680 | 13.5500 | 8.4700 | 0.006900 |
| 1.8789 | 57.3391 | 12.8192 | 15.6300 | 8.8100 | 0.258300 |
| 1.8278 | 58.2556 | 10.6506 | 14.3600 | 8.3900 | 0.138300 |
| 2.5134 | 67.2773 | 11.6880 | 15.7200 | 9.4900 | 0.056000 |
| 1.1840 | 55.5881 | 13.4008 | 16.3900 | 8.9900 | 0.177100 |
| 1.8311 | 76.6808 | 11.3257 | 16.4900 | 7.7250 | 0.110000 |
| 1.8634 | 75.0230 | 11.3991 | 12.8800 | 9.9700 | 0.069800 |
| 1.8335 | 80.5258 | 13.5388 | 17.5000 | 10.4300 | 0.257500 |
| 1.8178 | 65.1780 | 14.0528 | 16.6400 | 8.9000 | 0.083900 |
| 1.6686 | 90.7181 | 10.6691 | 15.5000 | 8.0200 | 0.069500 |
| 1.7527 | 67.2467 | 12.0962 | 13.4700 | 10.3700 | 0.093700 |
| 1.8135 | 82.0981 | 12.1009 | 15.2000 | 9.3200 | 0.037700 |
| 1.5032 | 98.8812 | 9.5360 | 11.9900 | 7.7030 | 0.003400 |
| 1.2437 | 96.6911 | 7.9160 | 12.7700 | 5.9500 | 0.242900 |
| 2.5498 | 88.4567 | 11.2064 | 14.6200 | 9.6900 | 0.126300 |
| 1.8910 | 49.2172 | 16.5953 | 19.1400 | 12.9100 | 0.002100 |
| 2.2148 | 61.9761 | 13.2166 | 16.0300 | 8.7900 | 0.001500 |
| 2.1650 | 64.0244 | 11.2667 | 13.2800 | 9.8200 | 0.307700 |
| 2.9305 | 98.6734 | 8.4872 | 9.7600 | 7.0370 | 0.336700 |
| 2.1119 | 68.3705 | 13.5956 | 15.8700 | 9.3100 | 0.051900 |
| 1.7025 | 78.2403 | 13.4551 | 18.3700 | 10.9700 | 0.061600 |
| 2.3981 | 75.2316 | 13.0791 | 15.7100 | 11.4400 | 0.162500 |
| 3.1479 | 64.9503 | 13.9684 | 16.0300 | 11.9100 | 0.012900 |
| 1.7389 | 67.0216 | 14.2466 | 16.8700 | 11.2800 | 0.008300 |
| 1.6991 | 79.0253 | 12.3994 | 13.9800 | 11.2300 | 0.056600 |
| 1.9621 | 85.8881 | 12.1206 | 16.1400 | 9.6200 | 0.102000 |
| 3.1333 | 57.3828 | 12.1298 | 13.7300 | 9.7500 | 0.005800 |
| 2.5599 | 97.6119 | 10.2947 | 14.2700 | 8.8200 | 0.026200 |
| 1.1337 | 99.9984 | 10.0511 | 11.1800 | 8.7500 | 0.207100 |
| 2.1214 | 98.0242 | 11.3348 | 15.4600 | 9.8500 | 0.086400 |
| 1.0166 | 99.9906 | 8.9288 | 11.4300 | 7.0990 | 0.094600 |
| 1.3488 | 99.9750 | 8.0279 | 8.8400 | 7.1930 | 0.405900 |
| 2.5983 | 65.4263 | 10.4535 | 15.1300 | 6.7460 | 0.165200 |
| 2.2801 | 40.9027 | 14.0916 | 16.7300 | 8.0400 | 0.016900 |
| 1.6377 | 66.0128 | 11.0170 | 17.1300 | 7.5710 | 0.115100 |
| 2.3636 | 86.3381 | 10.4073 | 13.5900 | 8.0700 | 0.086100 |
| 1.6133 | 97.3141 | 7.4392 | 9.3800 | 6.0770 | 0.291500 |
| 1.3486 | 92.4491 | 6.9779 | 11.7600 | 4.8220 | 0.388600 |
| 2.6887 | 78.6575 | 8.7544 | 11.7900 | 7.7300 | 0.032300 |
| 2.3805 | 62.7581 | 14.2216 | 17.5000 | 10.3900 | 0.014600 |
| 1.8142 | 61.6664 | 14.1261 | 19.7700 | 6.9650 | 0.335000 |
| 1.8281 | 66.0042 | 12.0312 | 14.0900 | 11.2100 | 0.303100 |
| 1.6760 | 66.6409 | 13.9394 | 18.9300 | 11.6400 | 0.002900 |
| 2.3900 | 58.7962 | 16.5920 | 19.2800 | 13.3200 | 0.062500 |
| 1.9741 | 66.8527 | 14.9756 | 19.9300 | 10.9300 | 0.332800 |
| 2.7934 | 96.5219 | 10.7063 | 14.2500 | 8.9500 | 0.042400 |
| 1.7936 | 92.2802 | 13.1878 | 17.2600 | 10.8200 | 0.038600 |
| 2.4240 | 99.8563 | 9.1275 | 10.5400 | 8.3200 | 0.259000 |
| 2.2580 | 99.9523 | 8.9815 | 12.9100 | 8.1900 | 0.226700 |
| 1.7088 | 80.2264 | 10.3398 | 11.6400 | 8.6800 | 0.046000 |
| 2.0536 | 56.9537 | 16.7705 | 20.0600 | 12.7800 | 0.014300 |
| 2.8555 | 64.4803 | 14.7048 | 19.0500 | 10.9000 | 0.385300 |
| 1.7042 | 70.6467 | 13.2194 | 16.1500 | 11.0900 | 0.382500 |
| 1.7851 | 72.7558 | 11.5033 | 13.3200 | 9.4400 | 0.016400 |
| 2.7008 | 46.3161 | 16.8936 | 19.1000 | 13.4000 | 0.063600 |
| 2.2232 | 56.3295 | 15.4367 | 21.3500 | 8.5900 | 0.143500 |
| 1.6745 | 85.8766 | 12.5119 | 14.3400 | 11.0100 | 0.216000 |
| 2.9562 | 61.3838 | 16.0145 | 18.9900 | 13.5800 | 0.020400 |
| 3.6341 | 47.6245 | 16.7033 | 21.4400 | 13.7600 | 0.377200 |
| 3.8493 | 74.2134 | 12.5459 | 15.2100 | 10.3700 | 0.206100 |
| 2.3944 | 63.1177 | 16.2502 | 20.0200 | 11.3800 | 0.247000 |
| 1.6041 | 53.2728 | 14.2892 | 21.0900 | 10.4800 | 0.365100 |
| 2.3041 | 73.5000 | 14.6759 | 17.4200 | 11.0800 | 0.120300 |
| 2.2319 | 50.9503 | 20.7219 | 25.1900 | 16.4800 | 0.001500 |
| 1.7756 | 54.3620 | 20.7097 | 24.7000 | 13.4500 | 0.161900 |
| 1.5434 | 76.7794 | 17.7664 | 24.0500 | 15.5900 | 0.389300 |
| 3.0400 | 77.1617 | 19.2498 | 22.4800 | 16.0500 | 0.057900 |
| 3.6663 | 43.3173 | 23.5413 | 28.5400 | 18.8400 | 0.031000 |
| 2.9546 | 72.9236 | 17.3459 | 23.4300 | 11.6400 | 0.373500 |
| 2.1185 | 57.3655 | 15.0183 | 18.7100 | 13.6100 | 0.352700 |
| 2.6469 | 79.1472 | 16.3798 | 21.4100 | 13.9800 | 0.013700 |
| 2.3017 | 41.6697 | 21.1903 | 23.1700 | 17.8500 | 0.089300 |
| 2.0661 | 42.5702 | 19.7422 | 24.7300 | 14.4700 | 0.247500 |
| 2.2130 | 41.0108 | 18.1564 | 20.6600 | 16.1700 | 0.556000 |
| 3.0946 | 44.7067 | 19.4136 | 25.0900 | 15.6300 | 0.061400 |
| 1.8086 | 51.5005 | 20.0834 | 22.8300 | 17.6700 | 0.001500 |
| 2.2527 | 41.8491 | 23.3962 | 26.1600 | 17.4800 | 0.337800 |
| 2.3350 | 53.7822 | 19.6239 | 24.5800 | 15.1900 | 0.361900 |
| 2.3848 | 56.7850 | 16.6602 | 19.4700 | 14.3800 | 0.087200 |
| 2.8267 | 45.6709 | 22.1602 | 25.1100 | 18.3400 | 0.027600 |
| 2.3967 | 59.8772 | 20.5288 | 24.2300 | 14.1400 | 0.323600 |
| 2.0800 | 68.9995 | 18.4283 | 22.7100 | 16.2800 | 0.334000 |
| 1.9810 | 49.7734 | 21.8083 | 25.8700 | 19.0900 | 0.035500 |
| 2.6870 | 40.4798 | 25.6269 | 30.2800 | 20.5900 | 0.086000 |
| 3.6223 | 53.7170 | 20.3255 | 25.2200 | 15.7500 | 0.536400 |
| 2.3791 | 61.1370 | 15.3619 | 18.3400 | 13.3800 | 0.375900 |
| 2.6228 | 50.8747 | 21.0148 | 26.0900 | 18.3000 | 0.005000 |
| 3.2888 | 31.5680 | 24.0981 | 25.7700 | 21.6800 | 0.170500 |
| 1.5549 | 36.9877 | 21.4948 | 27.0500 | 16.6700 | 0.438800 |
| 1.9558 | 45.0762 | 19.8227 | 21.7500 | 17.9900 | 0.074600 |
| 2.7385 | 35.0264 | 24.3998 | 28.3500 | 21.0400 | 0.170500 |
| 2.1137 | 38.3427 | 25.4123 | 28.1700 | 20.0200 | 0.211600 |
| 3.1301 | 54.6661 | 18.3119 | 21.2900 | 15.8100 | 0.502400 |
| 2.2364 | 56.1423 | 16.7372 | 17.7000 | 15.6300 | 0.074700 |
| 2.1652 | 29.4608 | 24.7009 | 27.5700 | 20.5000 | 0.003400 |
| 1.9441 | 25.6017 | 26.0114 | 29.6800 | 19.2000 | 0.354300 |
| 2.1535 | 35.9494 | 20.9873 | 22.5400 | 19.7800 | 0.175600 |
| 2.1041 | 46.3762 | 20.1177 | 23.6500 | 17.8300 | 0.055600 |
| 2.7082 | 45.0042 | 22.1863 | 24.0600 | 17.3400 | 0.043900 |
| 1.9870 | 65.1658 | 18.8866 | 21.2700 | 16.1500 | 0.499700 |
| 2.3580 | 90.4189 | 14.6138 | 18.8300 | 13.0800 | 0.465500 |
| 2.3233 | 53.9027 | 19.2763 | 24.0600 | 16.6300 | 0.030500 |
| 1.9518 | 27.7989 | 25.4039 | 27.4100 | 22.6500 | 0.099400 |
| 1.6011 | 39.2000 | 23.1472 | 28.3100 | 17.1900 | 0.554200 |
| 1.6753 | 53.4219 | 20.4172 | 22.6100 | 18.3500 | 0.005000 |
| 2.5061 | 45.1972 | 23.7314 | 29.2100 | 18.9700 | 0.006000 |
| 2.2780 | 36.2073 | 27.2292 | 29.5900 | 23.1700 | 0.109400 |
| 1.9870 | 65.1658 | 18.8866 | 21.2700 | 16.1500 | 0.499700 |
| 2.7082 | 45.0042 | 22.1863 | 24.0600 | 17.3400 | 0.043900 |
| 2.1137 | 38.3427 | 25.4123 | 28.1700 | 20.0200 | 0.211600 |
